# Two Years Later: Journals Are Not Yet Enforcing the ARRIVE Guidelines on Reporting Standards for Pre-Clinical Animal Studies

**DOI:** 10.1371/journal.pbio.1001756

**Published:** 2014-01-07

**Authors:** David Baker, Katie Lidster, Ana Sottomayor, Sandra Amor

**Affiliations:** 1 Blizard Institute, Barts and The London School of Medicine and Dentistry, Queen Mary University of London, London, United Kingdom; 2 Escola de Ciências da Saúde, Universidade do Minho, Braga, Portugal; 3 Pathology Department, VU University Medical Centre, Amsterdam, The Netherlands; University of California Davis, United States of America

## Abstract

There is growing concern that poor experimental design and lack of transparent reporting contribute to the frequent failure of pre-clinical animal studies to translate into treatments for human disease. In 2010, the Animal Research: Reporting of *In Vivo* Experiments (ARRIVE) guidelines were introduced to help improve reporting standards. They were published in *PLOS Biology* and endorsed by funding agencies and publishers and their journals, including PLOS, Nature research journals, and other top-tier journals. Yet our analysis of papers published in PLOS and Nature journals indicates that there has been very little improvement in reporting standards since then. This suggests that authors, referees, and editors generally are ignoring guidelines, and the editorial endorsement is yet to be effectively implemented.

## Abstract 2

A study by David Baker and colleagues reveals poor quality of reporting in pre-clinical animal research and a failure of journals to implement the ARRIVE guidelines.

## Introduction

Pre-clinical animal models of human neurological disease have delivered relatively few treatments [1],[2]. Despite reports of over 1,000 treatments effective in animal models of multiple sclerosis (MS), very few treatments have so far made it to the marketplace following initial development in disease-related animal models [2]. Similarly, in the case of stroke treatments, essentially no pre-clinical research has translated for human benefit [1]. What's worse, some treatments that ameliorate autoimmunity in animals, such as gamma interferon and tumour necrosis factor–specific antibodies, may exacerbate disease in humans [3]–[6]. The reasons why drugs that look promising in animal studies fail to translate into drug treatments for human disease include the following: issues with animals studies, such as the use of excessive doses and a timing of drug delivery that does not reflect that applied in established human disease [2],[7]; issues with clinical studies, such as the use of immunosuppressive drugs in progressive MS at a stage that is no longer responsive to peripheral immunosuppression [8]; and issues related to commercial interests, such as a lack of patent protection that provides no incentive for clinical development.

One important issue with animal studies is the widespread lack of transparent, quality reporting of study design and implementation [1],[2],[9]. Recent analyses have found, for example, that 86%–87% of papers reporting animal studies did not describe randomisation and blinding methods, and more than 95% of them did not report on the statistical power of the studies to detect a difference between experimental groups [2],[9]. This undermines the credibility of pre-clinical animal research. Inadequate reporting of key aspects of experimental design may reduce the impact of studies and could act as a barrier to translation by preventing repetition or inclusion in meta-analysis.

In June 2010, *PLOS Biology* published guidelines for reporting of experiments with animals [10]. The Animal Research: Reporting of *In Vivo* Experiments (ARRIVE) guidelines were drawn up by a group of statisticians, funders, and editors on the initiative of the UK National Centre for the Replacement, Refinement and Reduction of Animals in Research to improve consistency in reporting, notably, of pre-clinical animal studies. The ARRIVE guidelines consist of a 20-item checklist and recommendations for authors on reporting study design, experimental procedures, and experimental animals [10]. The ARRIVE guidelines are similar to the CONSORT (Consolidated Standards of Reporting Trials) statement required for reporting human clinical trials, which were introduced to alleviate inadequate reporting. Over 300 research journals (including those published by the Nature Publishing Group, PLOS, and BioMed Central) have endorsed the ARRIVE guidelines. So too have the major UK funding agencies (including the Wellcome Trust, the Biotechnology and Biological Sciences Research Council, and the Medical Research Council) and learned societies; the ARRIVE guidelines also form part of the US National Research Council Institute for Laboratory Animal Research guidance for the description of animal research in scientific publications [11]. Despite these good intentions, however, the ARRIVE guidelines are not being implemented by authors, reviewers, and journal editors [12]–[14]. Following an initial study to monitor the implementation and reporting of one specific statistical analysis in experimental design (see Text S1), we investigated the general adequacy of reporting on animal models of MS, a neuroimmunological disorder. Our survey of the literature uncovers worrying inadequacies in the reporting of experimental design, selecting appropriate statistical analyses, and applying key points in the ARRIVE guidelines.

## Lies, Damn Lies, and Statistics

Experimental autoimmune encephalomyelitis (EAE) in rodents is the principal model used to study the neurological and autoimmune mechanisms of MS in particular and autoimmunity in general. Rodents with EAE respond rapidly to drugs, and obvious clinical signs, such as limb paralysis, can be used to deduce underlying inflammatory aspects of the disease [7], so researchers can avoid the extensive tissue sampling and pathology tests required in other animal models. This ease of monitoring clinical disease and the responsiveness of the affected animals to drugs make the EAE model very amenable to drug testing. The clinical signs in animals are recorded using a subjective, non-linear motor-disability scale similar to the Kurtzke Expanded Disability Status Scale (EDSS) used to monitor MS in humans [15]. The severity of symptoms is scored numerically—usually as tail and limb paresis (i.e., partial paralysis), and sometimes as erection of the hair [2]—and the numerical score can then be used in statistical analysis. The degree of inflammation and the clinical scores reflecting ascending paresis of the limbs [15],[16] are clearly related; however, their relationship is non-linear.

Most researchers, in our opinion, make a fundamental error when reporting their scoring results: they use descriptive statistics, such as means and standard deviations, that assume the data are continuous, normally distributed, and of equal variance, and then apply parametric statistical tests that assume a specific population distribution for the data (such as ANOVA, *t*-tests, or regression analysis) to test the significance of their findings [13],[17]. Medians and ranges, which are perhaps more statistically appropriate, may not have the visual impact of a simple factor measuring differences between two treatment groups, and they lack the descriptive power of means and deviations [7]. Nevertheless, monitoring of treatment effects should be analysed using non-parametric statistical tests that make no assumptions about population distributions (such as the Mann–Whitney *U* test or Kruskall–Wallace test) to compare treatment groups when the data derive from arbitrary scale measurements, such as the motor-disability scale used in the EAE model; assuming a specific population, as is done for parametric statistics, is not appropriate [13],[17]. Although statistical arguments may be made for the use of parametric statistics on non-parametric data [6],[17], in the EAE literature a large variety of statistical approaches are currently being applied to test essentially the same hypothesis of a difference in outcome for a drug or gene manipulation treatment measured with the same non-linear, subjective assays.

## Are You Applying the Wrong Statistics?

**Figure 1:**
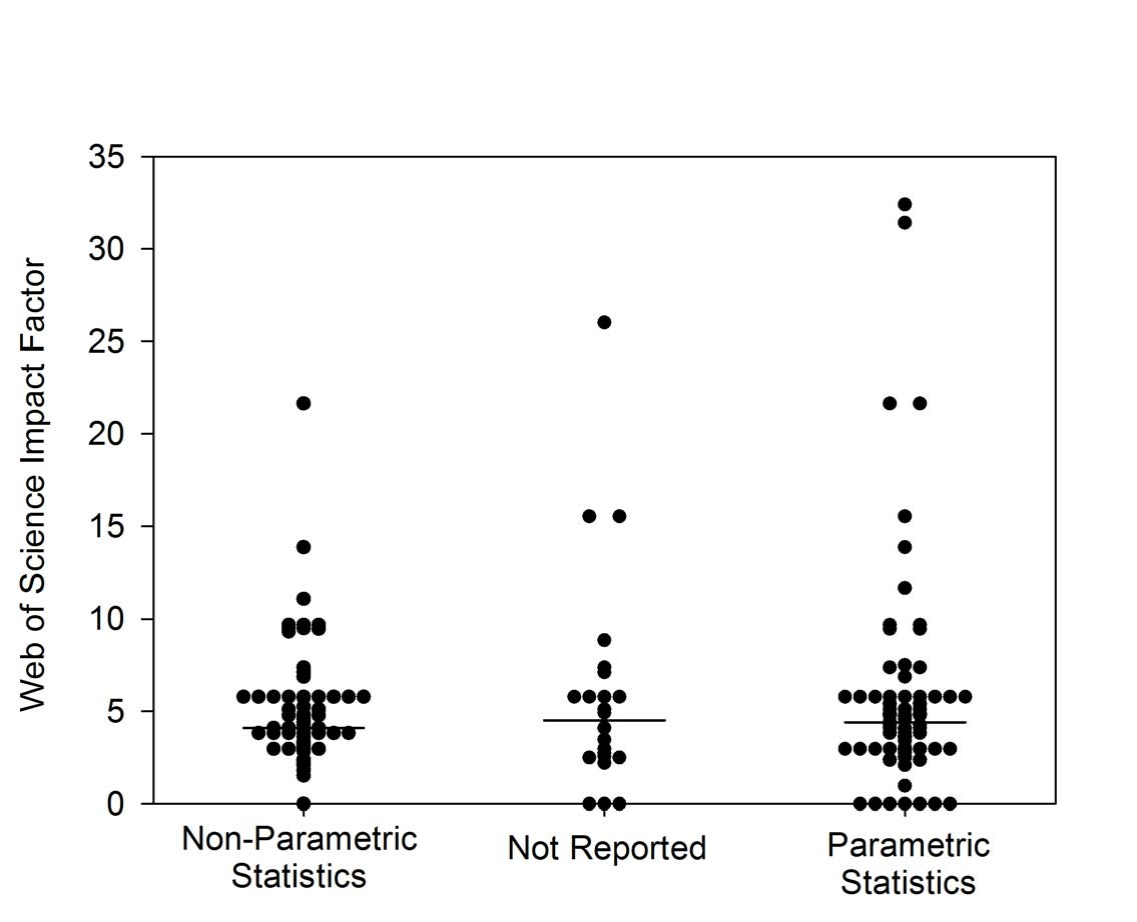
Inappropriate use of parametric statistics applied to non-parametric data in comparisons of treatments for EAE. Papers reporting differences between groups of animals with EAE were assessed to determine whether the studies reported the statistical analysis method, and whether they used non-parametric or parametric statistics to analyse non-parametric neurological scoring data (*n* = 152). Each publication was attributed an impact score according to the 2011 Web of Science impact factor for each journal. Some journals did not yet have an impact factor; papers in these journals were assigned an impact score of zero. The horizontal line shows the median impact score.

We analysed 180 primary papers archived in PubMed over a six-month period that compared EAE scores in two or more groups of animals (part 1 in Text S1; Table S1) to assess whether parametric tests or non-parametric tests were applied to experiments that tested the same hypothesis with very similar datasets [17]. We adopted the debatable position that non-parametric statistics should be applied to clinical disease. Thirteen percent (95% confidence interval [CI] 8.7%–18.5%) of articles did not report statistical analyses at all, and only 39% (95% CI 32.5%–46.8%) correctly used non-parametric statistical tests on non-parametric neurological scoring data. As many as 55% (95% CI 46.7%–62.3%) of studies, however, included analyses based on what we consider to be inappropriate statistical tests, and we saw no consistency in statistical tests of essentially the same hypothesis (part 2 in Text S1). The inappropriate use of statistics was independent of the impact factor of the journal in which the paper was published (Figure 1). This shows that reporting of inappropriate statistics occurs throughout the range of high- and low-impact-factor publications. Indeed, in journals that had an impact factor greater than ten, almost twice as many papers used incorrect statistics or failed to report statistics (10/107; 95% CI 5.2%–16.4%) as reported statistics correctly (3/69; 95% CI 1.5%–12.0%).

This observation led us to study papers on EAE published in several Nature journals, *Science*, *Cell*, and other top-ranking journals over two years (part 3 in Text S1; Table S2). Only 4% of EAE papers in these top-ranking journals (1/26; 95% CI 0.7%–18.9%) reported adequate use of a single non-parametric analysis of data on neurological scores, and 67% (95% CI 41.7%–84.8%) used only a *t*-test, which is not statistically justified [17]. Possibly some studies reporting inappropriate statistical methods were corrected during the peer-review process; however, this survey demonstrates significant weakness in the peer-review process and inconsistencies in reporting and statistical accuracy even between articles in the same journal. Most studies on EAE published during this period appeared in the *Journal of Immunology* (*n = *23) and the *Journal of Neuroimmunology* (*n = *13), in which adequate non-parametric statistics were reported in 39% and 31% of cases, respectively.

Non-parametric statistics will tend to approximate to parametric statistics when large group sizes are used; however, studies of EAE and most other animal models [2],[9] typically have small sample sizes, a limited scale size, and lack of appropriate “power/sample size calculations” (which ensure that there is a sufficient sample size in the experimental design to detect an effect of treatment, if there is one). In such cases, the chances of type I errors (i.e., false positives) against a null hypothesis of no treatment effect are enhanced, and type I errors probably occur. Consequently, these studies overestimate the benefit of the treatment. Consultation with an expert statistician to select an appropriate and valid test will minimise the chances not only of type I errors but also of type II errors (i.e., false negatives), which would fail to identify effective treatments.

Ensuring the use of appropriate statistical analysis is a common problem in many fields of biology [17]–[20]. Our survey suggests that the “high quality” journals are setting a poor standard for others to follow [19],[21]. While focussing on technically challenging and innovative science, many journals fail to ensure that the basic standards of experimental design and data analysis are adhered to. One solution to this problem is to have additional statistical review of submitted manuscripts (as is often done by journals in the health sciences); also, learned societies might suggest methods of analysis of standard outcomes and data reporting to their members [7],[12],[13].

## Are the Guidelines Being Ignored?

The ARRIVE guidelines lay out standards for reporting in all sections of published articles: the introduction (the background and objectives of the study), the methods (an ethical statement, description of the study design, experimental procedures and animals, housing and husbandry, sample size, and statistical methods), the results (numbers analysed and adverse events), the discussion (interpretation of the data, their implications, and potential for translation), and the acknowledgments. Given our findings of poor experimental design related to the use of appropriate statistics as outlined in the ARRIVE guidelines, we investigated whether other key aspects of the guidelines were being implemented.

**Figure 2:**
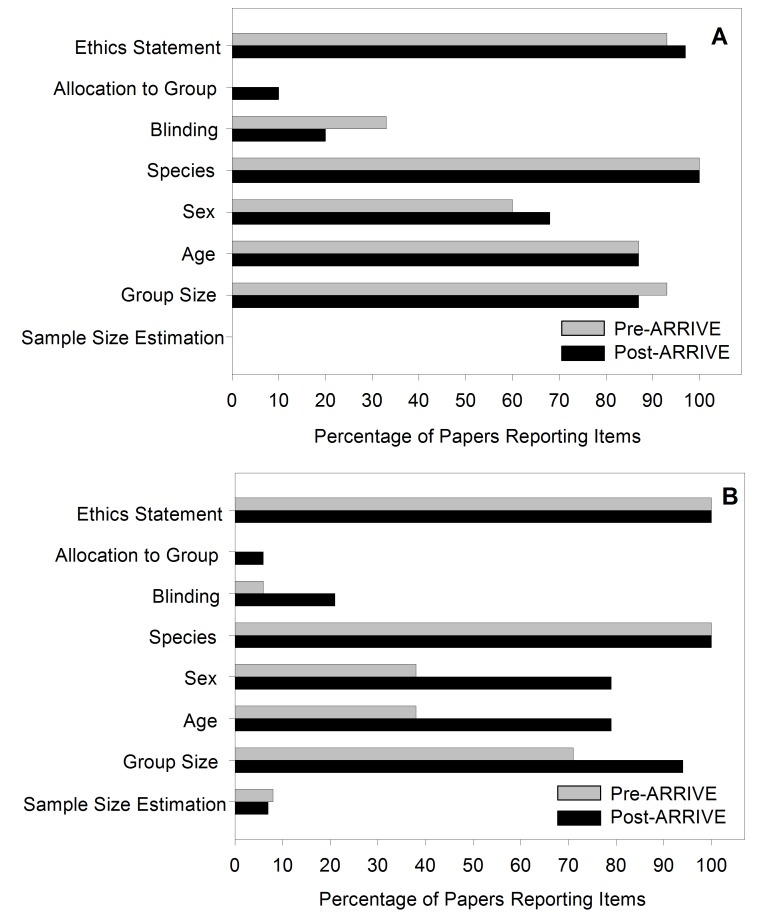
Impact of endorsement of ARRIVE guidelines on reporting of EAE studies in PLOS and Nature journals. Papers reporting differences between groups of animals with EAE were assessed over the two years before and the two years after the endorsement of the ARRIVE guidelines. The data show reporting of various aspects of experimental design in (A) PLOS (*n = *46) and (B) Nature journals (*n = *30).

We conducted another literature search for papers published during the two years before and two years after endorsement of the ARRIVE guidelines by all Nature and PLOS journals (Text S1; Figure 2). Many papers reported studies of EAE both before (*n = *15, PLOS journals; *n = *15, Nature journals) and after (*n = *30, PLOS journals, nearly all in *PLOS ONE*; *n = *14, Nature journals) publication of the ARRIVE guidelines (Table S3). We evaluated the articles in four key areas: ethics (whether there was ethical oversight and approval for the study via an institutional review), study design (allocation to groups/randomisation and blinding), experimental animals (species, sex, age, and group size), and sample size estimation/power calculations. We did not assess all 20 recommendations of the guidelines, because previous studies have suggested that very few papers fully incorporate them all [14].

Journals now commonly request ethical review statements, which featured in most papers in PLOS journals (93% pre-ARRIVE and 94% post-ARRIVE), Nature journals (100% pre-ARRIVE and 100% post-ARRIVE), and other journals [2]. Methods to reduce bias and the chance of false-positive reporting, by contrast, were rarely reported, although this does not mean they were not part of the experimental design [1],[2],[10]. We found that the percentage of studies, in the two years after endorsement of the ARRIVE guidelines, reporting blinding in their experimental design was similar to that in past surveys (20% in PLOS journals and 21% in Nature journals); however, fewer than 10% of the relevant studies in either Nature or PLOS journals reported randomisation (10% in PLOS journals and 0% in Nature journals), and even fewer mentioned any power/sample size analysis (0% in PLOS journals and 7% in Nature journals). Animal characteristics (species, sex, and age) and the number of animals used in a study can potentially influence experimental outcomes. We found an increase in the incidence of reporting of species (100% in both PLOS and Nature journals), sex (68% in PLOS journals and 79% in Nature journals), and age of animals (87% in PLOS journals and 79% in Nature journals) following publication of the ARRIVE guidelines. Not all papers reported this simple information, however (Figure 2). Reporting of statistical analysis was common, but, as mentioned above, use of parametric statistics on non-parametric data was the norm in EAE experiments both before and after endorsement of the ARRIVE guidelines; in fact, application of non-parametric statistics to neurological score data occurred less often in Nature journals after publication of the guidelines than before (25% pre-ARRIVE versus 7% post-ARRIVE).

Some of the studies examined here may have been designed before the introduction of the ARRIVE guidelines, but this should not have precluded appropriate reporting had the journals adopted the standards set out in the guidelines and provided the space to document this information. The possibility of publishing supplementary information online makes any argument about space limitation unfounded. Our findings suggest that, despite their endorsement by these journals, the guidelines have had little impact on reporting standards in published papers, at least in the neuroimmunological field, but the problem is likely to be more widespread [1],[2],[7],[16]. Evidence suggests that problems of analysis, design, and reporting apply to pre-clinical animal modelling throughout neuroscience and more generally in all areas of biological research [1],[2],[10],[14],[22]. Indeed, our findings on randomisation and blinding (Figure 2) are similar to those of a previous survey analysing 500 papers for generalised biology [10].

## How Might Journals Improve Reporting?

Fully implementing every aspect of the ARRIVE guidelines is clearly outside the current reporting norms in biology [7],[14] and seems unlikely to occur without a major change in the publication process. Endorsements of the ARRIVE guidelines are meaningless unless the signatories actually intend to implement them. The standard practice now to include reporting of ethical approval obtained before publication is one example where editorial action and a change in reporting behaviour has made a positive change: the majority of studies report on this now, compared to low levels of reporting a few years ago [2]. This demonstrates that it is feasible to implement certain reporting standards.

In response to claims that several publications in Nature journals contained irreproducible findings, the publisher introduced an editorial measure on 1 May 2013 to ensure that all papers published in Nature journals include key methodological details [23]. Authors must now submit a reporting checklist alongside manuscripts. In addition, Nature journals have removed space restrictions on the methods sections of their papers to allow authors to describe studies comprehensively. Some journals we looked at (12/169 in January 2013) and all PLOS journals except *PLOS ONE* (in December 2012) had yet to incorporate any requirements to use the ARRIVE guidelines when reporting into their instructions to authors. It seems essential for all journals not only to state their position on the ARRIVE guidelines, but also to give clear guidance to authors on how they should be applied and then to implement a policy of monitoring to document compliance [24],[25].

Some aspects of the ARRIVE guidelines, such as justification of selection of species and strain of animal used and the route and timing of delivery of agents [10], often form part of the ethical review process, which is currently being reported [2],[10], so there is no need to repeat this information in a paper. Similarly, it would be tedious to read the same justification for why mice were used in each paper in a journal that publishes mainly work on mice. Clinical studies are more diverse than mouse studies in their selection of patients, still in many pre-clinical studies the same methodology is used time and time again. A pragmatic approach might be to implement the most important aspects of the guidelines [3],[4], such as reporting the extent of blinding and randomisation [2],[10],[11]. Likewise, in clinical trials sample size/power calculations are important to limit false-negative findings, whereas this is rarely reported in animal studies that are invariably positive [1],[2],[26].

For journals such as *PLOS Medicine* and *PLOS Biology* that publish very few articles describing comparisons of treatment effects in vivo in animals, it would be relatively easy for editors to scrutinise the reporting in these papers. *PLOS ONE* currently publishes over 20,000 articles a year, however, so the scrutinising task must fall to the referees, who are clearly paying little attention at the moment to this aspect of the peer-review process. Factors they might consider that may impact the suitability of a study for publication include side effects of drugs, which may be apparent if specifically looked for [27],[28], the presence of infections in animals bought from commercial breeders, common defects in vision, hearing, etc., in lab mouse strains such as C57BL/6, BALB/c, and CBA/J [29],[30], and small sample size [1],[2],[10]. Lack of reporting may be because there is a publication bias toward reporting positive results [31],[32]. The review process might be better employed to assess the statistics being applied in an attempt to limit the publication of false-positive results. This approach could improve the potential for translation, as it would reduce the number of ineffective drugs being tested in the clinic for humans [2].

There may be a regional influence in the adoption of the ARRIVE guidelines, which were generated in the United Kingdom and were initially adopted by UK-based organisations. None of the senior authors of papers in our analysis were from UK-based laboratories, perhaps explaining their unfamiliarity with the guidelines. The guidelines have now been published in international journals and form part of recommendations made by the US National Research Council Institute for Laboratory Animal Research [11],[12], however, and ultimately, it remains the responsibility of the journal to enforce their application.

## Can ARRIVE Be Even More Human?

Recently, Gillman and colleagues suggested in *PLOS Biology* that the ARRIVE guidelines should be even more like guidelines for human randomised controlled trials, which require public registration of studies before they are performed [33]. This may be impractical, however, because animal studies often involve not a single experiment, as in a clinical trial, but a series of experiments that may evolve sometimes over a number of years. Public registration of experiments would also require a change in the patenting process, which often requires non-disclosure of the invention for patent validity. In addition, the results from animal experiments are crucial when filing patents. Changes to the requirements for reporting of animal experiments within patents might achieve the desired effect of giving translational animal studies transparency if they are to be used to support drug development for humans. The patent process does not currently have the perceived rigor of the peer-review process, as patents are judged from a legal perspective, but a consistent reporting standard could easily be adopted. This would require government support, but it would be in the public interest to uphold high-quality reporting standards. As universities want to exploit the inventions of their scientists, there would also be an incentive to adopt common reporting standards for the publishing and patenting worlds. As an initial step, the priority is that researchers adopt core elements of quality experimental design and reporting [12],[13].

## Supporting Information

Table S1 **Search results for statistical analysis of EAE data.** Results of a PubMed search using the term “experimental encephalomyelitis” during a six-month time period between 1 December 2011 and 31 May 2012.
(DOC)

Table S2 **Search results for statistical analysis of EAE data in high-impact-factor journals.** Results of a PubMed search using the term “experimental encephalomyelitis” during a time period between 1 January 2010 and 31 August 2012 in Nature journals, *Cell*, and *Science*.
(DOC)

Table S3 **Search results for analysis of reporting outcomes in EAE publications.** Results of a PubMed search using the term “experimental encephalomyelitis” during a two-year period for PLOS journals before (29 June 2008–28 June 2010) and after (29 June 2010–28 June 2012) endorsement of the ARRIVE guidelines, and for Nature journals before (1 February 2009–31 January 2011) and after (1 February 2011–31 January 2013) endorsement of the ARRIVE guidelines.
(DOC)

Text S1 **Supplementary methods and results.** Search methods, statistical analysis, and reporting outcomes.
(DOC)

## Abbreviations

95% CI: 95% confidence interval
ARRIVE: Animal Research: Reporting of *In Vivo* Experiments
EAE: experimental autoimmune encephalomyelitis
MS: multiple sclerosis
