# Supplementary material for: Two Years Later: Journals Are Not Yet Enforcing the ARRIVE Guidelines on Reporting Standards for Pre-Clinical Animal Studies
Source: PLoS Biol. 2014 Jan 7;12(1):e1001756. doi: 10.1371/journal.pbio.1001756 (PMC3883646; doi:10.1371/journal.pbio.1001756)
Supplement: Table S1 — Search results for statistical analysis of EAE data. Results of a PubMed search using the term “experimental encephalomyelitis” during a six-month time period between 1 December 2011 and 31 May 2012. (DOC) [file pbio.1001756.s001.doc]

**Supplementary Table 1**

1: Fonseca-Kelly Z, Nassrallah M, Uribe J, Khan RS, Dine K, Dutt M, Shindler KS. Resveratrol neuroprotection in a chronic mouse model of multiple sclerosis. Front Neurol. 2012;3:84. Epub 2012 May 24.

2: Downer EJ, Clifford E, Amu S, Fallon PG, Moynagh PN. The synthetic cannabinoid R(+)WIN55,212-2 augments interferon-β expression via peroxisome proliferator-activated receptor-α. J Biol Chem. 2012 May 31.

3: Fransson M, Piras E, Burman J, Nilsson B, Essand M, Lu B, Harris RA, Magnusson PU, Brittebo E, Loskog AS. CAR/FoxP3-engineered T regulatory cells target the CNS and suppress EAE upon intranasal delivery. J Neuroinflammation. 2012 May 30;9(1):112.

4: Gergely P, Nuesslein-Hildesheim B, Guerini D, Brinkmann V, Traebert M, Bruns C, et al. The selective S1P receptor modulator BAF312 redirects lymphocyte distribution and has species-specific effects on heart rate: translation from preclinical to clinical studies. Br J Pharmacol. 2012 May 30. doi: 10.1111/j.1476-5381.2012.02061.x.

5: Nakagome K, Matsushita S, Nagata M. Neutrophilic inflammation in severe asthma.Int Arch Allergy Immunol. 2012;158 Suppl 1:96-102. Epub 2012 May 15. PubMed PMID: 22627375.

6: Lyons A, Downer EJ, Costello DA, Murphy N, Lynch MA. Dok2 mediates the CD200Fc attenuation of Abeta-induced changes in glia. J Neuroinflammation. 2012 May 29;9(1):107. [Epub ahead of print] PubMed PMID: 22642833.

7: Solati J, Asiaei M, Hoseini MH. Using experimental autoimmune encephalomyelitis as a model to study the effect of prenatal stress on fetal programming. Neurol Res. 2012 May 30. [Epub ahead of print]

8: Yao SQ, Li ZZ, Huang QY, Li F, Wang ZW, Augusto E, He JC, Wang XT, Chen JF, Zheng RY. Genetic inactivation of the adenosine A(2A) receptor exacerbates brain damage in mice with EAE. J Neurochem. 2012 May 28. doi: 10.1111/j.1471-4159.2012.07807.x. [Epub ahead of print]

9: Fisher-Shoval Y, Barhum Y, Sadan O, Yust-Katz S, Ben-Zur T, Lev N, Benkler C, Hod M, Melamed E, Offen D. Transplantation of Placenta-Derived Mesenchymal Stem Cells in the EAE Mouse Model of MS. J Mol Neurosci. 2012 May 26. [Epub ahead of print] PubMed PMID: 22638856.

10: Tarcha EJ, Chi V, Munoz-Elias EJ, Bailey D, Londono LM, Upadhyay SK, Norton et al. Durable pharmacological responses from the peptide drug ShK-186, a specific Kv1.3 channel inhibitor that suppresses T cell mediators of autoimmune disease. J Pharmacol Exp Ther. 2012 May 25. [Epub ahead of print] PubMed PMID: 22637724

11: Namiki K, Matsunaga H, Yoshioka K, Tanaka K, Murata K, Ishida J, Sakairi A, et al. Mechanism for p38α-mediated experimental autoimmune encephalomyelitis. J Biol Chem. 2012 May 25. [Epub ahead of print] PubMed PMID: 22637476.

12: Yoshida R, Suzuki M, Sakaguchi R, Hasegawa E, Kimura A, Shichita T, Sekiya T, Shiraishi H, Shimoda K, Yoshimura A. Forced expression of stabilized c-Fos in dendritic cells reduces cytokine production and immune responses in vivo. Biochem Biophys Res Commun. 2012 May 24. [Epub ahead of print] PubMed PMID: 22634314.

13: Martinez NE, Sato F, Omura S, Minagar A, Alexander JS, Tsunoda I.Immunopathological patterns from EAE and Theiler's virus infection: Is multiple sclerosis a homogenous 1-stage or heterogenous 2-stage disease? Pathophysiology. 2012 May 25. [Epub ahead of print] PubMed PMID: 22633747.

14: Inoue M, Williams KL, Oliver T, Vandenabeele P, Rajan JV, Miao EA, Shinohara ML.Interferon-β Therapy Against EAE Is Effective Only When Development of the Disease Depends on the NLRP3 Inflammasome. Sci Signal. 2012 May 22;5(225):ra38. PubMed PMID: 22623753.

15: Nam Y, Choi M, Hwang H, Lee MG, Kwon BM, Lee WH, Suk K. Natural Flavone Jaceosidin is a Neuroinflammation Inhibitor. Phytother Res. 2012 May 23. doi: 10.1002/ptr.4737. [Epub ahead of print] PubMed PMID: 22619052

16: Chang RA, Miller SD, Longnecker R. Epstein-Barr virus latent membrane protein 2A exacerbates experimental autoimmune encephalomyelitis and enhances antigen presentation function. Sci Rep. 2012;2:353. Epub 2012 Apr 3. PubMed PMID:

17: Zettl UK, Patejdl R.Animal Models in Neurology: Drawbacks and Opportunities. Curr Pharm Des. 2012 May 16. [Epub ahead of print] PubMed PMID: 22612747. REVIEW

18: Reichardt HM, Lühder F. The Ambivalent Role of Apoptosis in Experimental Autoimmune Encephalomyelitis and Multiple Sclerosis. Curr Pharm Des. 2012 May 16.[Epub ahead of print] PubMed PMID: 22612741. REVIEW

19: Bai L, Lennon DP, Caplan AI, Dechant A, Hecker J, Kranso J, Zaremba A, Miller RH. Hepatocyte growth factor mediates mesenchymal stem cell-induced recovery in multiple sclerosis models. Nat Neurosci. 2012 May 20. doi: 10.1038/nn.3109. [Epub ahead of print] PubMed PMID: 22610068.

20: Wang Y, Marling SJ, Zhu JG, Severson KS, Deluca HF. Development of experimental autoimmune encephalomyelitis (EAE) in mice requires vitamin D and the vitamin D receptor. Proc Natl Acad Sci U S A. 2012 May 29;109(22):8501-4.

21: Zhou W, Dowell DR, Huckabee MM, Newcomb DC, Boswell MG, Goleniewska K, Lotz MT, Toki S, et al.Peebles RS Jr. Prostaglandin i(2) signaling drives th17 differentiation and exacerbates experimental autoimmune encephalomyelitis. PLoS One. 2012;7(5):e33518. Epub 2012 May 10. PubMed PMID: 22590492; PubMed Central PMCID: PMC3349674.

22: Mayer MC, Meinl E. Glycoproteins as targets of autoantibodies in CNS inflammation: MOG and more. Ther Adv Neurol Disord. 2012 May;5(3):147-59. PubMed PMID: 22590479; PubMed Central PMCID: PMC3349079.

23: Bergman CM, Marta CB, Maric M, Pfeiffer SE, Cresswell P, Ruddle NH. A Switch in Pathogenic Mechanism in Myelin Oligodendrocyte Glycoprotein-Induced Experimental Autoimmune Encephalomyelitis in IFN-γ-Inducible Lysosomal Thiol Reductase-Free Mice. J Immunol. 2012 Jun 15;188(12):6001-9. Epub 2012 May 14.

24: Kwon MJ, Ma J, Ding Y, Wang R, Sun Z. Protein Kinase C-θ Promotes Th17 Differentiation via Upregulation of Stat3. J Immunol. 2012 Jun 15;188(12):5887-97. Epub 2012 May 14. PubMed PMID: 22586032.

25: Jiang HR, Milovanović M, Allan D, Niedbala W, Besnard AG, Fukada SY, Alves-Filho JC, Togbe D, Goodyear CS, Linington C, Xu D, Lukic ML, Yew Liew F. IL-33 attenuates experimental autoimmune encephalomyelitis by suppressing IL-17 and IFN-γ production and inducing alternatively-activated macrophages. Eur J Immunol. 2012 May 14. doi: 10.1002/eji.201141947. [Epub ahead of print] PubMed

26: van der Star BJ, Vogel DV, Kipp M, Puentes F, Baker D, Amor S. In Vitro and In Vivo Models of Multiple Sclerosis.CNS Neurol Disord Drug Targets. 2012 May 11. [Epub ahead of print] PubMed PMID: 22583443. REVIEW

27: Gocke AR, Lebson LA, Grishkan IV, Hu L, Nguyen HM, Whartenby KA, Chandy KG, Calabresi PA. Kv1.3 Deletion Biases T Cells toward an Immunoregulatory Phenotype and Renders Mice Resistant to Autoimmune Encephalomyelitis. J Immunol. 2012 Jun 15;188(12):5877-86. Epub 2012 May 11.

28: Chen X, Ma L, Jiang Y, Chen S, Zhu C, Liu M, Ma X, Zhu D, Liu Y, Peng F, WangQ, Pi R. Minocycline up-regulates the expression of brain-derived neurotrophic factor and nerve growth factor in experimental autoimmune encephalomyelitis. Eur J Pharmacol. 2012 Jul 5;686(1-3):124-9. Epub 2012 May 2. PubMed PMID: 22575526.

29: Payne N, Dantanarayana A, Sun G, Moussa L, Caine S, McDonald C, Herszfeld D, Bernard CC, Siatskas C. Early intervention with gene-modified mesenchymal stem cells overexpressing interleukin-4 enhances anti-inflammatory responses and etc.Cell Adh Migr. 2012 May 1;6(3). [Epub ahead of print] PubMed PMID: 22568986.

30: Kim do Y, Hao J, Liu R, Turner G, Shi FD, Rho JM. Inflammation-mediated memory dysfunction and effects of a ketogenic diet in a murine model of multiple sclerosis. PLoS One. 2012;7(5):e35476. Epub 2012 May 2. PubMed PMID: 22567104;

31: Prinz M, Knobeloch KP. Type I interferons as ambiguous modulators of chronic inflammation in the central nervous system. Front Immunol. 2012;3:67. Epub 2012 Apr 9. PubMed PMID: 22566948; PubMed Central PMCID:

32: Passani MB, Ballerini C. Histamine and neuroinflammation: insights from murine experimental autoimmune encephalomyelitis. Front Syst Neurosci. 2012;6:32. Epub 2012 May 3.

33: Shindler KS, Revere K, Dutt M, Ying GS, Chung DC. In vivo detection ofexperimental optic neuritis by pupillometry. Exp Eye Res. 2012 Apr 24;100C:1-6. [Epub ahead of print] PubMed PMID: 22561341.

34: Jukkola PI, Lovett-Racke AE, Zamvil SS, Gu C. K(+) channel alterations in the progression of experimental autoimmune encephalomyelitis.Neurobiol Dis. 2012 Aug;47(2):280-93. Epub 2012 Apr 24

35: Kandagaddala LD, Kang MJ, Haque MM, Im HY, Seo JE, Chung BC, Jung BH,Patterson TA, Kwon OS. In vitro screening of NADPH oxidase inhibitors and in vivo effects of L-leucinethiol on EAE-induced mice. J Neurol Sci. 2012 Jul 15;318(1-2):36-44. Epub 2012 May 2. PubMed PMID:22554692.

36: Yadav V, Bourdette DN, Bowen JD, Lynch SG, Mattson D, Preiningerova J, Bever CT Jr, Simon J, Goldstein A, Burrows GG, Offner H, Ferro AJ, Vandenbark AA. Recombinant T-Cell Receptor Ligand (RTL) for Treatment of Multiple Sclerosis: A Double-Blind, Placebo-Controlled, Phase 1, Dose-Escalation Study. Autoimmune Dis. 2012;2012:954739. Epub 2012 Apr 5. PubMed PMID: 22548151; PubMed Central PMCID: PMC3328144.

37: Barr TA, Shen P, Brown S, Lampropoulou V, Roch T, Lawrie S, Fan B, O'Connor RA, Anderton SM, Bar-Or A, Fillatreau S, Gray D. B cell depletion therapy ameliorates autoimmune disease through ablation of IL-6-producing B cells. J Exp Med. 2012 May 7;209(5):1001-10. Epub 2012 Apr 30. PubMed PMID: 22547654; PubMed Central PMCID: PMC3348102

38: Yuan S, Shi Y, Tang SJ. Wnt Signaling in the Pathogenesis of Multiple Sclerosis-Associated Chronic Pain. J Neuroimmune Pharmacol. 2012 May 2. [Epubahead of print] PubMed PMID: 22547300.

39: Schiffmann S, Ferreiros N, Birod K, Eberle M, Schreiber Y, Pfeilschifter W, Ziemann U, Pierre S, Scholich K, Grösch S, Geisslinger G. Ceramide synthase 6 plays a critical role in the development of experimental autoimmune encephalomyelitis. J Immunol. 2012 Jun 1;188(11):5723-33. Epub 2012 Apr 27. PubMed PMID: 22544924.

40: Petratos S, Ozturk E, Azari MF, Kenny R, Young Lee J, Magee KA, Harvey AR, McDonald C, Taghian K, et al.Limiting multiple sclerosis related axonopathy by blocking Nogo receptor and CRMP-2 phosphorylation. Brain. 2012 Jun;135(Pt 6):1794-818. Epub 2012 Apr 28. PubMed PMID: 22544872.

41: Abourbeh G, Thézé B, Maroy R, Dubois A, Brulon V, Fontyn Y, Dollé F, Tavitian B, Boisgard R. Imaging microglial/macrophage activation in spinal cords of EAE by positron emission tomography using the mitochondrial 18 kDa translocator protein J Neurosci. 2012 Apr 25;32(17):5728-36. PubMed PMID: 22539835.

42: Maine CJ, Hamilton-Williams EE, Cheung J, Stanford SM, Bottini N, Wicker LS, Sherman LA. PTPN22 Alters the Development of Regulatory T Cells in the Thymus. J Immunol. 2012 Jun 1;188(11):5267-75. Epub 2012 Apr 25. PubMed PMID: 22539785;

43: Hoehlig K, Shen P, Lampropoulou V, Roch T, Malissen B, O'Connor R, Ries S, Hilgenberg E, Anderton SM, Fillatreau S. Activation of CD4⁺ Foxp3⁺ regulatory T cells proceeds normally in the absence of B cells during EAE. Eur J Immunol. 2012May;42(5):1164-73. doi: 10.1002/eji.201142242. PubMed PMID: 22539290.

44: Segal BM. The unwavering commitment of regulatory T cells in the suppression of autoimmune encephalomyelitis: another aspect of immune privilege in the CNS. Eur J Immunol. 2012 May;42(5):1102-5. doi: 0.1002/eji.201242567. PubMed PMID: 22539285.

45: Ma J, Wang R, Fang X, Sun Z. β-Catenin/TCF-1 Pathway in T Cell Development and Differentiation. J Neuroimmune Pharmacol. 2012 Apr 27. [Epub ahead of print] PubMed PMID: 22535304.

46: Geffard M, Duleu S, Bessede A, Vigier V, Bodet D, Mangas A, Coveñas R. GEMSP: A New Therapeutic Approach to Multiple Sclerosis. Cent Nerv Syst Agents Med Chem.2012 Apr 25. [Epub].

47: Vanheel A, Daniels R, Plaisance S, Baeten K, Hendriks JJ, Leprince P, Dumont D, Robben J, Brône B, Stinissen P, Noben JP, Hellings N. Identification of protein networks involved in the disease course of experimental autoimmune encephalomyelitis, an animal model of multiple sclerosis.PLoS One.2012;7(4):e35544. Epub 2012 Apr 17. PubMed PMID: 22530047; PubMed Central PMCID: PMC3328452.

48: Wu M, Nissen JC, Chen EI, Tsirka SE. Tuftsin promotes an anti-inflammatory switch and attenuates symptoms in experimental autoimmune encephalomyelitis.PLoS One. 2012;7(4):e34933. Epub 2012 Apr 17. PubMed PMID: 22529957; PubMed Central PMCID: PMC3328491.

49: Mills JH, Kim DG, Krenz A, Chen JF, Bynoe MS. A2A Adenosine Receptor Signaling in Lymphocytes and the Central Nervous System Regulates Inflammation during Experimental Autoimmune Encephalomyelitis. J Immunol. 2012 Jun 1;188(11):5713-22. Epub 2012 Apr 23. PubMed PMID: 22529293; PubMed Central PMCID PMC3358473.

50: Yang J, Yan Y, Ma CG, Kang T, Zhang N, Gran B, Xu H, Li K, Ciric B, Zangaladze A, Curtis M, Rostami A, Zhang GX. Accelerated and enhanced effect of CCR5-transduced bone marrow neural stem cells on autoimmune encephalomyelitis.Acta Neuropathol. 2012 Apr 22. [Epub ahead of print] PubMed PMID: 22526024.

51: Ziehn MO, Avedisian AA, Dervin SM, O'Dell TJ, Voskuhl RR. Estriol preserves synaptic transmission in the hippocampus during autoimmune demyelinating disease.Lab Invest. 2012 Apr 23. doi: 10.1038/labinvest.2012.76.

52: Wang J, Chen F, Zheng P, Deng W, Yuan J, Peng B, Wang R, Liu W, Zhao H, Wang Y, Wu G. Huperzine A ameliorates experimental autoimmune encephalomyelitis via the suppression of T cell-mediated neuronal inflammation in mice. Exp Neurol. 2012 Jul;236(1):79-87. Epub 2012 Apr 15. PubMed PMID: 22524989.

53: Craner MJ, Fugger L. Misfiring in multiple sclerosis: cerebellar channelopathy, a potential novel target? Ann Neurol. 2012 Apr;71(4):437-8. doi: 10.1002/ana.23537. PubMed PMID: 22522437.

54: Fitzgerald DC, Zhang GX, Yu S, Cullimore ML, Zhao Z, Rostami A. Intravenous tolerance effectively overcomes enhanced pro-inflammatory responses and EAE severity in the absence of IL-12 receptor signaling. J Neuroimmunol. 2012 Jun 15;247(1-2):32-7. Epub 2012 Apr 21.

55: Mycko MP, Cichalewska M, Machlanska A, Cwiklinska H, Mariasiewicz M, Selmaj KW. Micro RNA-301a regulation of a T-helper 17 immune response controls autoimmune demyelination.Proc Natl Acad Sci U S A. 2012 May 15;109(20):E1248-57. Epub 2012

56: Li M, Li Y, Liu X, Gao X, Wang Y. IL-33 blockade suppresses the development of experimental autoimmune encephalomyelitis in C57BL/6 mice. J Neuroimmunol. 2012 Jun 15;247(1-2):25-31. Epub 2012 Apr 18. PubMed PMID:

57: Payne NL, Sun G, Herszfeld D, Tat-Goh PA, Verma PJ, Parkington HC, Coleman HA, Tonta MA, Siatskas C, Bernard CC. Comparative study on the therapeutic potential of neurally differentiated stem cells in a mouse model of multiple sclerosis.PLoS One. 2012;7(4):e35093. Epub 2012 Apr 13. PubMed PMID: 22514711;

58: Rice J. Animal models: Not close enough. Nature. 2012 Apr 12;484(7393):S9. PubMed PMID: 22509510. REVIEW

59: Ljubisavljevic S, Stojanovic I, Pavlovic D, Milojkovic M, Vojinovic S, Sokolovic D, Stevanovic I. Correlation of nitric oxide levels in the cerebellum and spinal cord of experimental autoimmune encephalomyelitis rats with clinical symptoms. Acta Neurobiol Exp (Wars). 2012;72(1):33-9. PubMed PMID: 22508082.

60: Shaw MK, Zhao XQ, Tse HY. Overcoming unresponsiveness in experimental autoimmune encephalomyelitis (EAE) resistant mouse strains by adoptive transfer and antigenic challenge. J Vis Exp. 2012 Apr 9;(62):e3778. doi: 10.3791/3778.

61: Ljubisavljevic S, Stojanovic I, Pavlovic R, Sokolovic D, Pavlovic D, Cvetkovic T, Stevanovic I. Modulation of nitric oxide synthase by arginase and methylated arginines during the acute phase of experimental multiple sclerosis. J Neurol Sci. 2012 Jul 15;318(1-2):106-11. Epub 2012 Apr 15. PubMed PMID: 22507752.

62: Elyaman W, Bassil R, Bradshaw EM, Orent W, Lahoud Y, Zhu B, Radtke F, Yagita H, Khoury SJ. Notch receptors and Smad3 signaling cooperate in the induction of interleukin-9-producing T cells. Immunity. 2012 Apr 0; 36(4):623-34. Epub 2012

63: Gan Y, Liu R, Wu W, Bomprezzi R, Shi FD. Antibody to α4 integrin suppresses natural killer cells infiltration in central nervous system in experimental autoimmune encephalomyelitis. J Neuroimmunol. 2012 Jun 15;247(1-2):9-15. Epub 2012 Apr 13. PubMed PMID: 22503411; PubMed Central PMCID: PMC3351567.

64: Loda E, Balabanov R. Interferon regulatory factor 1 regulation of oligodendrocyte injury and inflammatory demyelination. Rev Neurosci. 2012 Jan 26;23(2):145-52. doi: 10.1515/revneuro-2011-068.

65: Berard JL, Zarruk JG, Arbour N, Prat A, Yong VW, Jacques FH, Akira S, David S. Lipocalin 2 is a novel immune mediator of experimental autoimmune encephalomyelitis pathogenesis and is modulated in multiple sclerosis.

Glia. 2012 Jul;60(7):1145-59. doi: 10.1002/glia.22342. Epub 2012 Apr 12. PubMed PMID: 22499213.

66: Kim H, Walczak P, Muja N, Campanelli JT, Bulte JW. ICV-transplanted human glial precursor cells are short-lived yet exert immunomodulatory effects in mice with EAE. Glia. 2012 Jul;60(7):1117-29. doi: 10.1002/glia.22339. Epub 2012 Apr

67: Lee DH, Linker RA. The role of myelin oligodendrocyte glycoprotein in autoimmune demyelination: a target for multiple sclerosis therapy? Expert Opin Ther Targets. 2012 Apr 12. [Epub ahead of print] PubMed PMID: 22494461.

68: de Graaf KL, Albert M, Weissert R. Autoantigen conformation influences both B- and T-cell responses and encephalitogenicity. J Biol Chem. 2012 May 18;287(21):17206-13. Epub 2012 Apr 5. PubMed PMID: 22493449; PubMed Central PMCID: PMC3366859.

69: Nakatsuji Y, Okuno T, Moriya M, Sugimoto T, Kinoshita M, Takamatsu H, Nojima S, Kimura T, Kang S, Ito D, Nakagawa Y, Toyofuku et alElevation of Sema4A Implicates Th Cell Skewing and the Efficacy of IFN-β Therapy in Multiple Sclerosis. J Immunol. 2012 May 15;188(10):4858-65. Epub 2012 Apr 9. PubMed PMID: 22491253.

70: Weinger JG, Davies P, Acker CM, Brosnan CF, Tsiperson V, Bayewitz A, Shafit-Zagardo B. Mice devoid of Tau have increased susceptibility to neuronal damage in myelin oligodendrocyte glycoprotein-induced EAE.

J Neuropathol Exp Neurol. 2012 May;71(5):422-33. PubMed PMID:

71: Ahn M, Yang W, Kim H, Jin JK, Moon C, Shin T. Immunohistochemical study of arginase-1 in the spinal cords of Lewis rats with experimental autoimmune encephalomyelitis. Brain Res. 2012 May 9;1453:77-86. Epub 2012 Mar 16. PubMedPMID: 22483960.

72: Ebringer A, Rashid T, Wilson C. The role of Acinetobacter in the pathogenesis of multiple sclerosis examined by using Popper sequences. Med Hypotheses. 2012Jun;78(6):763-9. Epub 2012 Apr 5. PubMed PMID: 22483667.

73: Kuijk LM, Klaver EJ, Kooij G, van der Pol SM, Heijnen P, Bruijns SC, Kringel H, Pinelli E, Kraal G, de Vries HE, Dijkstra CD, Bouma G, van Die I. Soluble helminth products suppress clinical signs in murine EAE and differentially modulate human dendritic cell activation.Mol Immunol. 2012 Jun;51(2):210-8. Epub 2012 Apr 5. PubMed PMID: 22482518.

74: Agrawal SM, Silva C, Wang J, Tong JP, Yong VW. A novel anti-EMMPRIN function-blocking antibody reduces T cell proliferation and neurotoxicity: relevance to multiple sclerosis. J Neuroinflammation. 2012 Apr 5;9(1):64. [Epub ahead of print] PubMed PMID: 22480370.

75: Schulze-Topphoff U, Shetty A, Varrin-Doyer M, Molnarfi N, Sagan SA, Sobel RA, Nelson PA, Zamvil SS. Laquinimod, a quinoline-3-carboxamide, induces type II myeloid cells that modulate central nervous system autoimmunity. PLoS One. 2012;7(3):e33797. Epub 2012 Mar 30. PubMed PMID: 22479444; PubMed Central PMCID: PMC3316495.

76: Lee DH, Kubera K, Rosenthal B, Kaltschmidt B, Kaltschmidt C, Gold R, Linker RA.Neuronal NF-κB ablation does not influence neuro-axonal degeneration in experimental autoimmune demyelination.

J Neuroimmunol. 2012 May 15;246(1-2):38-42. Epub 2012 Apr 3. PubMed PMID: 22475633.

77: Sher F, Amor S, Gerritsen W, Baker D, Jackson SL, Boddeke E, Copray S.Intraventricularly injected Olig2-NSCs attenuate established relapsing-remitting EAE in mice. Cell Transplant. 2012 Mar 28. [Epub ahead of print] PubMed PMID: 22469520.

78: Oem JK, Yoon HJ, Kim HR, Roh IS, Lee KH, Lee OS, Bae YC. Genetic andpathogenic characterization of Akabane viruses isolated from cattle withencephalomyelitis in Korea. Vet Microbiol. 2012 Mar 1. [Epub ahead of print]

PubMed PMID: 22464491.

79: Markoullis K, Sargiannidou I, Gardner C, Hadjisavvas A, Reynolds R, KleopaKA. Disruption of oligodendrocyte gap junctions in experimental autoimmune encephalomyelitis. Glia. 2012 Jul;60(7):1053-66. doi: 10.1002/glia.22334. Epub 2012 Mar 27. PubMed PMID: 22461072.

80: Mangalam AK, Luckey D, Giri S, Smart M, Pease LR, Rodriguez M, David CS. Two discreet subsets of CD8 T cells modulate PLP(91-110) induced experimentalautoimmune encephalomyelitis in HLA-DR3 transgenic mice. J Autoimmun. 2012Jun;38(4):344-53. Epub 2012 Mar 28. PubMed PMID: 22459490.

81: Atkinson W, Forghani R, Wojtkiewicz GR, Pulli B, Iwamoto Y, Ueno T, Waterman P, Truelove J, Oklu R, Chen JW. Ligation of the jugular veins does not result in brain inflammation or demyelination in mice. PLoS One. 2012;7(3):e33671. Epub2012 Mar 21. PubMed PMID: 22457780; PubMed Central PMCID: PMC3310075.

82: Langer HF, Choi EY, Zhou H, Schleicher R, Chung KJ, Tang Z, Göbel K, Bdeir K,Chatzigeorgiou A, Wong C, Bhatia S, Kruhlak MJ, Rose JW, Burns JB, Hill KE, Qu H, Zhang Y, Lehrmann E, Becker KG, Wang Y, Simon DI, Nieswandt B, Lambris JD, Li X, Meuth SG, Kubes P, Chavakis T. Platelets contribute to the pathogenesis ofexperimental autoimmune encephalomyelitis. Circ Res. 2012 Apr 27;110(9):1202-10. Epub 2012 Mar 27. PubMed PMID: 22456181.

83: Noga MJ, Dane A, Shi S, Attali A, van Aken H, Suidgeest E, Tuinstra T,Muilwijk B, Coulier L, Luider T, Reijmers TH, Vreeken RJ, Hankemeier T.Metabolomics of cerebrospinal fluid reveals changes in the central nervous system

metabolism in a rat model of multiple sclerosis. Metabolomics. 2012Apr;8(2):253-263. Epub 2011 Apr 16. PubMed PMID: 22448154; PubMed Central PMCID: PMC3291832. Not comparing EAE groups

84: Hucke S, Floßdorf J, Grützke B, Dunay IR, Frenzel K, Jungverdorben J,Linnartz B, Mack M, Peitz M, Brüstle O, Kurts C, Klockgether T, Neumann H, Prinz M, Wiendl H, Knolle P, Klotz L. Licensing of myeloid cells promotes centralnervous system autoimmunity and is controlled by peroxisomeproliferator-activated receptor γ. Brain. 2012 May;135(Pt 5):1586-605. Epub 2012 Mar 24. PubMed PMID: 22447120.

85: Wheeler C, Nabors LB, Barnum S, Yang X, Hu X, Schoeb TR, Chen D, Ardelt AA,King PH. Sex hormone-dependent attenuation of EAE in a transgenic mouse withastrocytic expression of the RNA regulator HuR. J Neuroimmunol. 2012 May15;246(1-2):34-7. Epub 2012 Mar 22. PubMed PMID: 22445740; PubMed Central PMCID: PMC3335935.

86: Zhang HL, Azimullah S, Zheng XY, Wang XK, Amir N, Mensah-Brown EP, Al Shamsi M, Shahin A, Press R, Zhu J, Adem A. IFN-γ deficiency exacerbates experimentalautoimmune neuritis in mice despite a mitigated systemic Th1 immune response. JNeuroimmunol. 2012 May 15;246(1-2):18-26. Epub 2012 Mar 23. PubMed PMID:22445739.

87: Lescher J, Paap F, Schultz V, Redenbach L, Scheidt U, Rosewich H, Nessler S, Fuchs E, Gärtner J, Brück W, Junker A. MicroRNA regulation in experimentalautoimmune encephalomyelitis in mice and marmosets resembles regulation in human multiple sclerosis lesions. J Neuroimmunol. 2012 May 15;246(1-2):27-33. Epub 2012Mar 22. PubMed PMID: 22445295.

88: Mann MK, Ray A, Basu S, Karp CL, Dittel BN. Pathogenic and regulatory rolesfor B cells in experimental autoimmune encephalomyelitis. Autoimmunity. 2012 Apr 19. [Epub ahead of print] PubMed PMID: 22443691.

89: Sina C, Lipinski S, Gavrilova O, Aden K, Rehman A, Till A, Rittger A,Podschun R, Meyer-Hoffert U, Haesler R, Midtling E, Pütsep K, McGuckin MA,Schreiber S, Saftig P, Rosenstiel P. Extracellular cathepsin K exerts

antimicrobial activity and is protective against chronic intestinal inflammation in mice. Gut. 2012 Mar 22. [Epub ahead of print] PubMed PMID: 22442160.

90: Yuan XQ, Qiu G, Liu XJ, Liu S, Wu Y, Wang X, Lu T. Fluoxetine promotesremission in acute experimental autoimmune encephalomyelitis in rats.Neuroimmunomodulation. 2012;19(4):201-8. Epub 2012 Mar 21. PubMed PMID: 22441536.

91: Yu D, Corbett B, Yan Y, Zhang GX, Reinhart P, Cho SJ, Chin J. Earlycerebrovascular inflammation in a transgenic mouse model of Alzheimer's disease. Neurobiol Aging. 2012 Mar 20. [Epub ahead of print]

92: Forghani R, Wojtkiewicz GR, Zhang Y, Seeburg D, Bautz BR, Pulli B, MilewskiAR, Atkinson WL, Iwamoto Y, Zhang ER, Etzrodt M, Rodriguez E, Robbins CS, SwirskiFK, Weissleder R, Chen JW. Demyelinating diseases: myeloperoxidase as an imaging biomarker and therapeutic target. Radiology. 2012 May;263(2):451-60. Epub 2012Mar 21. PubMed PMID: 22438365; PubMed Central PMCID: PMC3329272.

93: Friedman-Levi Y, Hoftberger R, Budka H, Mayer-Sonnenfeld T, Abramsky O,Ovadia H, Gabizon R. Targeting of prion-infected lymphoid cells to the centralnervous system accelerates prion infection. J Neuroinflammation. 2012 Mar21;9:58. PubMed PMID: 22436404; PubMed Central PMCID: PMC3347999.

94: Müller AM, Jun E, Conlon H, Sadiq SA. Inhibition of SLPI ameliorates disease activity in experimental autoimmune encephalomyelitis. BMC Neurosci. 2012 Mar21;13:30. PubMed PMID: 22436018;.

95: Dalgas U, Stenager E. Exercise and disease progression in multiple sclerosis:can exercise slow down the progression of multiple sclerosis? Ther Adv NeurolDisord. 2012 Mar;5(2):81-95. PubMed PMID: 22435073;

96: Yan Y, Ding X, Li K, Ciric B, Wu S, Xu H, Gran B, Rostami A, Zhang GX.CNS-specific Therapy for Ongoing EAE by Silencing IL-17 Pathway in Astrocytes.Mol Ther. 2012 Mar 20. doi: 10.1038/mt.2012.12. [Epub ahead of print]

97: Kalinke U, Prinz M. Endogenous, or therapeutically induced, type I interferonresponses differentially modulate Th1/Th17-mediated autoimmunity in the CNS. Immunol Cell Biol. 2012 May;90(5):505-9

98: Meng Q, Zhao B, Xu Q, Xu X, Deng G, Li C, Luan L, Ren F, Wang H, Xu H, Xu Y, Zhang H, Xiang JN, Elliott JD, Guo TB, Zhao Y, Zhang W, Lu H, Lin X.Indole-propionic acid derivatives as potent, S1P3-sparing and EAE efficacious

sphingosine-1-phosphate 1 (S1P1) receptor agonists. Bioorg Med Chem Lett. 2012Apr 15;22(8):2794-7. Epub 2012 Mar 3. PubMed PMID: 22429468.

99. Waiczies H, Millward JM, Lepore S, Infante-Duarte C, Pohlmann A, Niendorf T, Waiczies S. Identification of cellular infiltrates during early stages of braininflammation with magnetic resonance microscopy. PLoS One. 2012;7(3):e32796. Epub 2012 Mar 12. PubMed PMID: 22427887; PubMed Central PMCID: PMC3299701.

100: Hampton DW, Innes N, Merkler D, Zhao C, Franklin RJ, Chandran S. Focal immune-mediated white matter demyelination reveals an age-associated increase in axonal vulnerability and decreased remyelination efficiency. Am J Pathol. 2012 May;180(5):1897-905. Epub 2012 Mar 15. PubMed PMID: 22426338.

101: Liu Y, Zhao H, Zhang J, Zhang P, Li M, Qi F, Wang Y, Kou S, Zheng Q, Wang LThe regulatory effect of liuwei dihuang pills on cytokines in mice withexperimental autoimmune encephalomyelitis. Am J Chin Med. 2012;40(2):295-308.PubMed PMID: 22419424.

102: Radosavljevic G, Volarevic V, Jovanovic I, Milovanovic M, Pejnovic N,Arsenijevic N, Hsu DK, Lukic ML. The roles of Galectin-3 in autoimmunity andtumor progression. Immunol Res. 2012 Apr;52(1-2):100-10.

103: Jun S, Ochoa-Repáraz J, Zlotkowska D, Hoyt T, Pascual DW. Bystander-mediatedstimulation of proteolipid protein-specific regulatory T (Treg) cells confersprotection against experimental autoimmune encephalomyelitis (EAE) via TGF-β. JNeuroimmunol. 2012 Apr;245(1-2):39-47. Epub 2012 Mar 13. PubMed PMID: 22418032;

104: Qin H, Yeh WI, De Sarno P, Holdbrooks AT, Liu Y, Muldowney MT, Reynolds SL, Yanagisawa LL, Fox TH 3rd, Park K, Harrington LE, Raman C, Benveniste EN. Signal transducer and activator of transcription-3/suppressor of cytokine signaling-3(STAT3/SOCS3) axis in myeloid cells regulates neuroinflammation. Proc Natl AcadSci U S A. 2012 Mar 27;109(13):5004-9. Epub 2012 Mar 12. PubMed PMID: 22411837;

105: Mackenzie-Graham AJ, Rinek GA, Avedisian A, Morales LB, Umeda E, Boulat B,Jacobs RE, Toga AW, Voskuhl RR. Estrogen treatment prevents gray matter atrophyin experimental autoimmune encephalomyelitis. J Neurosci Res. 2012Jul;90(7):1310-23. doi: 10.1002/jnr.23019. Epub 2012 Mar 13. PubMed PMID:22411609; PubMed Central PMCID: PMC3350614.

106: Nakamura T, Asano M, Sekiguchi Y, Mizuno Y, Tamaki K, Nara F, Kawase Y, YabeY, Nakai D, Kamiyama E, Urasaki-Kaneno Y, Shimozato T, Doi-Komuro H, Kagari T,Tomisato W, Inoue R, Nagasaki M, Yuita H, Oguchi-Oshima K, Kaneko R, Nishi T.Synthesis and evalution of CS-2100, a potent, orally active and S1P(3)- sparing S1P(1) agonist. Eur J Med Chem. 2012 May;51:92-8. Epub 2012 Feb 25. PubMed PMID: 22405291.

107: Kanakasabai S, Casalini E, Walline CC, Mo C, Chearwae W, Bright JJ.Differential regulation of CD4(+) T helper cell responses by curcumin inexperimental autoimmune encephalomyelitis. J Nutr Biochem. 2012 Mar 6. [Epub

ahead of print] PubMed PMID: 22402368.

108: Reekmans K, Praet J, De Vocht N, Daans J, Van der Linden A, Berneman Z,Ponsaerts P. Stem cell therapy for multiple sclerosis: preclinical evidencebeyond all doubt? Regen Med. 2012 Mar;7(2):245-59.

109: Hagemeyer N, Boretius S, Ott C, Von Streitberg A, Welpinghus H, Sperling S, Frahm J, Simons M, Ghezzi P, Ehrenreich H. Erythropoietin attenuates neurologicaland histological consequences of toxic demyelination in mice. Mol Med. 2012 Feb29. doi: 10.2119/molmed.2011.00457. [Epub ahead of print] PubMed PMID: 22396019.

110: Smolinska A, Posma JM, Blanchet L, Ampt KA, Attali A, Tuinstra T, Luider T, Doskocz M, Michiels PJ, Girard FC, Buydens LM, Wijmenga SS. Simultaneous analysisof plasma and CSF by NMR and hierarchical models fusion. Anal Bioanal Chem. 2012 May;403(4):947-59. Epub 2012 Mar 7. PubMed PMID: 22395451; PubMed Central PMCID: PMC3336062.

111: Chan KH, Zhang R, Kwan JS, Guo VY, Ho PW, Ho JW, Chu AC. Aquaporin-4autoantibodies cause asymptomatic aquaporin-4 loss and activate astrocytes inmouse. J Neuroimmunol. 2012 Apr;245(1-2):32-8. Epub 2012 Mar 5. PubMed PMID:22394609.

112: Colombo E, Cordiglieri C, Melli G, Newcombe J, Krumbholz M, Parada LF,Medico E, Hohlfeld R, Meinl E, Farina C. Stimulation of the neurotrophin receptorTrkB on astrocytes drives nitric oxide production and neurodegeneration. J ExpMed. 2012 Mar 12;209(3):521-35. Epub 2012 Mar 5. PubMed PMID: 22393127; PubMedCentral PMCID: PMC3302220.

113: Burrows GG, Meza-Romero R, Huan J, Sinha S, Mooney JL, Vandenbark AA, OffnerH. Gilt required for RTL550-CYS-MOG to treat experimental autoimmune encephalomyelitis. Metab Brain Dis. 2012 Jun;27(2):143-9. Epub 2012 Mar 7. PubMed PMID: 22392628; PubMed Central PMCID: PMC3348371.

114: Ilic N, Gruden-Movsesijan A, Sofronic-Milosavljevic L. Trichinella spiralis:shaping the immune response. Immunol Res. 2012 Apr;52(1-2):111-9. PubMed PMID:22392054.

115: Milovanovic M, Volarevic V, Radosavljevic G, Jovanovic I, Pejnovic N,Arsenijevic N, Lukic ML. IL-33/ST2 axis in inflammation and immunopathology.Immunol Res. 2012 Apr;52(1-2):89-99. PubMed PMID: 22392053.

116: Momcilović M, Mostarica-Stojković M, Miljković D. CXCL12 in control of neuroinflammation. Immunol Res. 2012 Apr;52(1-2):53-63. PubMed PMID: 22392052.

117: Severin IC, Souza AL, Davis JH, Musolino N, Mack M, Power CA, Proudfoot AE. Properties of 7ND-CCL2 are modulated upon fusion to Fc. Protein Eng Des Sel. 2012 May;25(5):213-22. Epub 2012 Mar 2.

118: Liu JQ, Liu Z, Zhang X, Shi Y, Talebian F, Carl JW Jr, Yu C, Shi FD,Whitacre CC, Trgovcich J, Bai XF. Increased Th17 and regulatory T cell responses in EBV-induced gene 3-deficient mice lead to marginally enhanced development of autoimmune encephalomyelitis. J Immunol. 2012 Apr 1;188(7):3099-106. Epub 2012 Mar 2. PubMed PMID: 22387555; PubMed Central PMCID: PMC3311737.

119: Redondo M, Brea J, Perez DI, Soteras I, Val C, Perez C, Morales-García JA,Alonso-Gil S, Paul-Fernandez N, Martin-Alvarez R, Cadavid MI, Loza MI,Perez-Castillo A, Mengod G, Campillo NE, Martinez A, Gil C. Effect of

phosphodiesterase 7 (PDE7) inhibitors in experimental autoimmune encephalomyelitis mice. Discovery of a new chemically diverse family of compounds. J Med Chem. 2012 Apr 12;55(7):3274-84. Epub 2012 Mar 16.

120: Prado C, Contreras F, González H, Díaz P, Elgueta D, Barrientos M, Herrada AA, Lladser Á, Bernales S, Pacheco R. Stimulation of dopamine receptor D5 expressed on dendritic cells potentiates Th17-mediated immunity. J Immunol. 2012 Apr 1;188(7):3062-70. Epub 2012 Feb 29. PubMed PMID: 22379034.

121: Büyüktimkin B, Wang Q, Kiptoo P, Stewart JM, Berkland C, Siahaan TJ. Vaccine-like controlled-release delivery of an immunomodulating peptide to treat experimental autoimmune encephalomyelitis. Mol Pharm. 2012 Apr 2;9(4):979-85.Epub 2012 Mar 13. PubMed PMID: 22375937; PubMed Central PMCID: PMC3357633.

122: Yi T, Song SU. Immunomodulatory properties of mesenchymal stem cells and their therapeutic applications. Arch Pharm Res. 2012 Feb;35(2):213-21. Epub 2012 Feb 28. PubMed PMID: 22370776.

123: Manikwar P, Büyüktimkin B, Kiptoo P, Badawi AH, Galeva NA, Williams TD,Siahaan TJ. I-domain-antigen conjugate (IDAC) for delivering antigenic peptides to APC: synthesis, characterization, and in vivo EAE suppression. Bioconjug Chem. 2012 Mar 21;23(3):509-17. Epub 2012 Mar 12. PubMed PMID: 22369638; PubMed CentralPMCID: PMC3311109.

124: Ray A, Basu S, Williams CB, Salzman NH, Dittel BN. A novel IL-10-independent regulatory role for B cells in suppressing autoimmunity by maintenance of regulatory T cells via GITR ligand. J Immunol. 2012 Apr 1;188(7):3188-98. Epub 2012 Feb 24. PubMed PMID: 22368274; PubMed Central PMCID: PMC3311743.

125: Shields SD, Cheng X, Gasser A, Saab CY, Tyrrell L, Eastman EM, Iwata M, Zwinger PJ, Black JA, Dib-Hajj SD, Waxman SG. A channelopathy contributes to cerebellar dysfunction in a model of multiple sclerosis. Ann Neurol. 2012 Feb;71(2):186-94. doi: 10.1002/ana.22665. PubMed PMID: 22367990.

126: Locatelli G, Wörtge S, Buch T, Ingold B, Frommer F, Sobottka B, Krüger M, Karram K, Bühlmann C, Bechmann I, Heppner FL, Waisman A, Becher B. Primary oligodendrocyte death does not elicit anti-CNS immunity. Nat Neurosci. 2012 Feb 26;15(4):543-50. doi: 10.1038/nn.3062. PubMed PMID: 22366759.

127: Dimitrijević M, Mitić K, Kuštrimović N, Vujić V, Stanojević S. NPY suppressed development of experimental autoimmune encephalomyelitis in Dark Agouti rats by disrupting costimulatory molecule interactions. J Neuroimmunol. 2012 Apr;245(1-2):23-31. Epub 2012 Feb 23. PubMed PMID: 22365383.

128: Dai H, Ciric B, Zhang GX, Rostami A. Interleukin-10 plays a crucial role in suppression of experimental autoimmune encephalomyelitis by Bowman-Birk inhibitor. J Neuroimmunol. 2012 Apr;245(1-2):1-7. Epub 2012 Feb 25. PubMed PMID: 22365083; PubMed Central PMCID: PMC3339487.

129: Kleinschnitz C, Meuth SG, Magnus T, Korn T, Linker RA; on behalf of the speakers at the 3'rd NEUROWIND e.V. scientific meeting. Report on the 3'rd scientific meeting of the "Verein zur Förderung des Wissenschaftlichen

Nachwuchses in der Neurologie" (NEUROWIND e.V.) held in Motzen, Germany, Nov. 4'th - Nov. 6'th, 2011. Exp Transl Stroke Med. 2012 Feb 23;4(1):2. PubMed PMID:22360825; PubMed Central PMCID: PMC3299621.

130: Sajic M, Hunt DP, Lee W, Compston DA, Schweimer JV, Gregson NA, Chandran S, Smith KJ. Mesenchymal stem cells lack efficacy in the treatment of experimentalautoimmune neuritis despite in vitro inhibition of T-cell proliferation. PLoSOne. 2012;7(2):e30708. Epub 2012 Feb 16. PubMed PMID: 22359549; PubMed CentralPMCID: PMC3281026. NOT EAE

131: Pareek TK, Belkadi A, Kesavapany S, Zaremba A, Loh SL, Bai L, Cohen ML,Meyer C, Liby KT, Miller RH, Sporn MB, Letterio JJ. Triterpenoid modulation ofIL-17 and Nrf-2 expression ameliorates neuroinflammation and promotesremyelination in autoimmune encephalomyelitis. Sci Rep. 2011;1:201. Epub 2011 Dec19. PubMed PMID: 22355716; PubMed Central PMCID: PMC3242013

132: Poppensieker K, Otte DM, Schürmann B, Limmer A, Dresing P, Drews E, Schumak B, Klotz L, Raasch J, Mildner A, Waisman A, Scheu S, Knolle P, Förster I, Prinz M, Maier W, Zimmer A, Alferink J. CC chemokine receptor 4 is required for experimental autoimmune encephalomyelitis by regulating GM-CSF and IL-23 production in dendritic cells. Proc Natl Acad Sci U S A. 2012 Mar 6;109(10):3897-902. Epub 2012 Feb 21.

133: Siatskas C, Seach N, Sun G, Emerson-Webber A, Silvain A, Toh BH, Alderuccio F, Bäckström BT, Boyd RL, Bernard CC. Thymic Gene Transfer of MyelinOligodendrocyte Glycoprotein Ameliorates the Onset but Not the Progression ofAutoimmune Demyelination. Mol Ther. 2012 Feb 21. doi: 10.1038/mt.2012.15. [Epubahead of print] PubMed PMID: 22354375.

134: Zheng J, Dasgupta A, Bizzozero OA. Changes in 20S subunit composition arelargely responsible for altered proteasomal activities in experimental autoimmune encephalomyelitis. J Neurochem. 2012 May;121(3):486-94. doi:

10.1111/j.1471-4159.2012.07699.x. Epub 2012 Mar 14. PubMed PMID: 22353035; PubMedCentral PMCID: PMC3323733.

135: Stoye D, Schubert C, Goihl A, Guttek K, Reinhold A, Brocke S, Grüngreiff K, Reinhold D. Zinc aspartate suppresses T cell activation in vitro and relapsingexperimental autoimmune encephalomyelitis in SJL/J mice. Biometals. 2012Jun;25(3):529-39. Epub 2012 Feb 19. PubMed PMID: 22350510.

136: Mandolesi G, Grasselli G, Musella A, Gentile A, Musumeci G, Sepman H, HajiN, Fresegna D, Bernardi G, Centonze D. GABAergic signaling and connectivity onPurkinje cells are impaired in experimental autoimmune encephalomyelitis.Neurobiol Dis. 2012 May;46(2):414-24. Epub 2012 Feb 12. PubMed PMID: 22349452.

137: Lukens JR, Barr MJ, Chaplin DD, Chi H, Kanneganti TD. Inflammasome-derivedIL-1β regulates the production of GM-CSF by CD4(+) T cells and γδ T cells. J Immunol. 2012 Apr 1;188(7):3107-15. Epub 2012 Feb 17. PubMed PMID: 22345669.

138: Herges K, de Jong BA, Kolkowitz I, Dunn C, Mandelbaum G, Ko RM, Maini A, Han MH, Killestein J, Polman C, Goodyear AL, Dunn J, Steinman L, Axtell RC. Protective effect of an elastase inhibitor in a neuromyelitis optica-like disease driven by a peptide of myelin oligodendroglial glycoprotein. Mult Scler. 2012 Apr;18(4):398-408. Epub 2012 Feb 16. PubMed PMID: 22343184; PubMed Central PMCID:PMC3319834.

139: de Lago E, Moreno-Martet M, Cabranes A, Ramos JA, Fernández-Ruiz J.Cannabinoids ameliorate disease progression in a model of multiple sclerosis in mice, acting preferentially through CB1 receptor-mediated anti-inflammatory effects. Neuropharmacology. 2012 Jun;62(7):2299-308. Epub 2012 Feb 8. PubMedPMID: 22342378.

140: Lutz SE, Raine CS, Brosnan CF. Loss of astrocyte connexins 43 and 30 doesnot significantly alter susceptibility or severity of acute experimentalautoimmune encephalomyelitis in mice. J Neuroimmunol. 2012 Apr;245(1-2):8-14.Epub 2012 Feb 18. PubMed PMID: 22342190.

141: Oomizu S, Arikawa T, Niki T, Kadowaki T, Ueno M, Nishi N, Yamauchi A,Hirashima M. Galectin-9 suppresses Th17 cell development in an IL-2-dependent butTim-3-independent manner. Clin Immunol. 2012 Apr;143(1):51-8. Epub 2012 Jan 17. PubMed PMID: 22341088.

142: Gupta AA, Ding D, Lee RK, Levy RB, Bhattacharya SK. Spontaneous ocular andneurologic deficits in transgenic mouse models of multiple sclerosis andnoninvasive investigative modalities: a review. Invest Ophthalmol Vis Sci. 2012Feb 13;53(2):712-24. Print 2012 Feb. Review. PubMed PMID: 22331505; PubMedCentral PMCID: PMC3317416 REVIEW.

143: Sonobe Y, Li H, Jin S, Kishida S, Kadomatsu K, Takeuchi H, Mizuno T,Suzumura A. Midkine inhibits inducible regulatory T cell differentiation bysuppressing the development of tolerogenic dendritic cells. J Immunol. 2012 Mar

15;188(6):2602-11. Epub 2012 Feb 8. PubMed PMID: 22323540.

144: Cossetti C, Alfaro-Cervello C, Donegà M, Tyzack G, Pluchino S. New perspectives of tissue remodelling with neural stem and progenitor cell-basedtherapies. Cell Tissue Res. 2012 Feb 10. [Epub ahead of print]

145: Rosenling T, Stoop MP, Attali A, van Aken H, Suidgeest E, Christin C, StinglC, Suits F, Horvatovich P, Hintzen RQ, Tuinstra T, Bischoff R, Luider TM.Profiling and identification of cerebrospinal fluid proteins in a rat EAE model

of multiple sclerosis. J Proteome Res. 2012 Apr 6;11(4):2048-60. Epub 2012 Feb23. PubMed PMID: 22320401.

146: Batoulis H, Uhl M, Addicks K, Lehmann PV, Kuerten S. The magnitude of theantigen-specific T cell response is separated from the severity of spinal cordhistopathology in remitting-relapsing experimental autoimmune encephalomyelitis. Glia. 2012 May;60(5):794-805. doi: 10.1002/glia.22309. Epub 2012 Feb 8. PubMedPMID: 22319015.

147: Rothbard JB, Kurnellas MP, Brownell S, Adams CM, Su L, Axtell RC, Chen R,Fathman CG, Robinson WH, Steinman L. Therapeutic effects of systemicadministration of chaperone αB-crystallin associated with binding proinflammatoryplasma proteins. J Biol Chem. 2012 Mar 23;287(13):9708-21. Epub 2012 Feb 3.PubMed PMID: 22308023; PubMed Central PMCID: PMC3322989.

148: Arima Y, Harada M, Kamimura D, Park JH, Kawano F, Yull FE, Kawamoto T,Iwakura Y, Betz UA, Márquez G, Blackwell TS, Ohira Y, Hirano T, Murakami M.Regional neural activation defines a gateway for autoreactive T cells to crossthe blood-brain barrier. Cell. 2012 Feb 3;148(3):447-57. PubMed PMID: 22304915.

149: Frigo M, Cogo MG, Fusco ML, Gardinetti M, Frigeni B. Glutamate and multiple sclerosis. Curr Med Chem. 2012;19(9):1295-9. PubMed PMID: 22304707.

150: Rodrigues DH, Vilela Mde C, Lacerda-Queiroz N, Miranda AS, Sousa LF, ReisHJ, Teixeira AL. Behavioral investigation of mice with experimental autoimmuneencephalomyelitis. Arq Neuropsiquiatr. 2011 Dec;69(6):938-42. PubMed PMID:22297884.

151: Kumar N, Lyda B, Chang MR, Lauer JL, Solt LA, Burris TP, Kamenecka TM,Griffin PR. Identification of SR2211: a potent synthetic RORγ-selectivemodulator. ACS Chem Biol. 2012 Apr 20;7(4):672-7. Epub 2012 Feb 13. PubMed PMID: 22292739; PubMed Central PMCID: PMC3331898.

152: Muili KA, Gopalakrishnan S, Meyer SL, Eells JT, Lyons JA. Amelioration ofexperimental autoimmune encephalomyelitis in C57BL/6 mice by photobiomodulationinduced by 670 nm light. PLoS One. 2012;7(1):e30655. Epub 2012 Jan 24. PubMedPMID: 22292010; PubMed Central PMCID: PMC3265499.

153: Oh DY, Cui P, Hosseini H, Mosse J, Toh BH, Chan J. Potentlyimmunosuppressive 5-fluorouracil-resistant mesenchymal stromal cells completelyremit an experimental autoimmune disease. J Immunol. 2012 Mar 1;188(5):2207-17. Epub 2012 Jan 30. PubMed PMID: 22291191.

154: Polak PE, Kalinin S, Braun D, Sharp A, Lin SX, Feinstein DL. The vincaminederivative vindeburnol provides benefit in a mouse model of multiple sclerosis:effects on the Locus coeruleus. J Neurochem. 2012 Apr;121(2):206-16. doi:10.1111/j.1471-4159.2012.07673.x. Epub 2012 Feb 17. PubMed PMID: 22288774.

155: O'Connor RA, Li X, Blumerman S, Anderton SM, Noelle RJ, Dalton DK. Adjuvant immunotherapy of experimental autoimmune encephalomyelitis: immature myeloid cells expressing CXCL10 and CXCL16 attract XCR3+CXCR6+ and myelin-specific Tcells to the draining lymph nodes rather than the central nervous system. JImmunol. 2012 Mar 1;188(5):2093-101. Epub 2012 Jan 27. PubMed PMID: 22287719.

156: Libbey JE, Cusick MF, Tsunoda I, Fujinami RS. Antiviral CD8⁺ T cells causean experimental autoimmune encephalomyelitis-like disease in naive mice. JNeurovirol. 2012 Feb;18(1):45-54. Epub 2012 Jan 27. PubMed PMID: 22281874; PubMedCentral PMCID: PMC3327473.

157: Gourdain P, Ballerini C, Nicot AB, Carnaud C. Exacerbation of experimentalautoimmune encephalomyelitis in prion protein (PrPc)-null mice: evidence for acritical role of the central nervous system. J Neuroinflammation. 2012 Jan26;9:25. PubMed PMID: 22281016; PubMed Central PMCID: PMC3305405.

158: Karlik SJ, Roscoe WA, Patinote C, Contino-Pepin C. Targeting vascularchanges in lesions in multiple sclerosis and experimental autoimmuneencephalomyelitis. Cent Nerv Syst Agents Med Chem. 2012 Mar;12(1):7-14. PubMedPMID: 22280405.

159: Anderson AC, Chandwaskar R, Lee DH, Sullivan JM, Solomon A,Rodriguez-Manzanet R, Greve B, Sobel RA, Kuchroo VK. A transgenic model ofcentral nervous system autoimmunity mediated by CD4+ and CD8+ T and B cells. JImmunol. 2012 Mar 1;188(5):2084-92. Epub 2012 Jan 25. PubMed PMID: 22279107;PubMed Central PMCID: PMC3288950.

160: Morando S, Vigo T, Esposito M, Casazza S, Novi G, Principato MC, Furlan R,Uccelli A. The therapeutic effect of mesenchymal stem cell transplantation inexperimental autoimmune encephalomyelitis is mediated by peripheral and centralmechanisms. Stem Cell Res Ther. 2012 Jan 26;3(1):3. PubMed PMID: 22277374; PubMedCentral PMCID: PMC3340547.

161: Su KG, Savino C, Marracci G, Chaudhary P, Yu X, Morris B, Galipeau D,Giorgio M, Forte M, Bourdette D. Genetic inactivation of the p66 isoform of ShcA is neuroprotective in a murine model of multiple sclerosis. Eur J Neurosci. 2012 Feb;35(4):562-71. doi: 10.1111/j.1460-9568.2011.07972.x. Epub 2012 Jan 25. PubMedPMID: 22277070; PubMed Central PMCID: PMC3279590.

162: Gnanapavan S, Grant D, Pryce G, Jackson S, Baker D, Giovannoni G.Neurofilament a biomarker of neurodegeneration in autoimmune encephalomyelitis.Autoimmunity. 2012 Jun;45(4):298-303. Epub 2012 Feb 15. PubMed PMID: 22276904.

163: Bielekova B, McFarland H. Comment on "Interleukin-2/interleukin-2 antibodytherapy induces target organ natural killer cells that inhibit central nervoussystem inflammation". Ann Neurol. 2012 Jan;71(1):148-9. doi: 10.1002/ana.22547.PubMed PMID: 22275261.

164: Roy M, Richard JF, Dumas A, Vallières L. CXCL1 can be regulated by IL-6 and promotes granulocyte adhesion to brain capillaries during bacterial toxinexposure and encephalomyelitis. J Neuroinflammation. 2012 Jan 23;9:18. PubMedPMID: 22269426; PubMed Central PMCID: PMC3283467.

165: Lisi L, Navarra P, Cirocchi R, Sharp A, Stigliano E, Feinstein DL, DelloRusso C. Rapamycin reduces clinical signs and neuropathic pain in a chronic modelof experimental autoimmune encephalomyelitis. J Neuroimmunol. 2012 Feb29;243(1-2):43-51. Epub 2012 Jan 20. PubMed PMID: 22264993.

166: Zhu B, Trikudanathan S, Zozulya AL, Sandoval-Garcia C, Kennedy JK, Atochina O, Norberg T, Castagner B, Seeberger P, Fabry Z, Harn D, Khoury SJ, Guleria I.Immune modulation by Lacto-N-fucopentaose III in experimental autoimmuneencephalomyelitis. Clin Immunol. 2012 Mar;142(3):351-61. Epub 2011 Dec 30. PubMedPMID: 22264636; PubMed Central PMCID: PMC3288504.

167: Koya V, Campbell AM, McLaughlin PJ, Tewari D, Wilson RP, Cooper TK. Outbreakof abdominal distension and obstipation in a C57BL/6J experimental autoimmuneencephalomyelitis study. Vet Pathol. 2012 May;49(3):528-31. Epub 2012 Jan 18.PubMed PMID: 22262350.

168: Quintanar-Stephano A, Organista-Esparza A, Chavira-Ramírez R, Kovacs K,Berczi I. Effects of neurointermediate pituitary lobectomy and desmopressin onacute experimental autoimmune encephalomyelitis in Lewis rats.

Neuroimmunomodulation. 2012;19(3):148-57. Epub 2012 Jan 18. PubMed PMID:22262014.

169: Ellrichmann G, Thöne J, Lee DH, Rupec RA, Gold R, Linker RA. Constitutiveactivity of NF-kappa B in myeloid cells drives pathogenicity of monocytes andmacrophages during autoimmune neuroinflammation. J Neuroinflammation. 2012 Jan20;9:15. PubMed PMID: 22260436; PubMed Central PMCID: PMC3274436.

170: Martín R, Hernández M, Córdova C, Nieto ML. Natural Triterpenes ModulateImmune-Inflammatory Hallmarks to Protect against Experimental AutoimmuneEncephalomyelitis. Therapeutic Implications for Multiple Sclerosis. Br JPharmacol. 2012 Jan 20. doi: 10.1111/j.1476-5381.2012.01869.x. [Epub ahead ofprint] PubMed PMID: 22260389.

171: Yoshida H, Kimura A, Fukaya T, Sekiya T, Morita R, Shichita T, Inoue H,Yoshimura A. Low dose CP-690,550 (tofacitinib), a pan-JAK inhibitor, acceleratesthe onset of experimental autoimmune encephalomyelitis by potentiating Th17differentiation. Biochem Biophys Res Commun. 2012 Feb 10;418(2):234-40. Epub 2012Jan 9. PubMed PMID: 22252297.

172: Sun JB, Czerkinsky C, Holmgren J. B lymphocytes treated in vitro withantigen coupled to cholera toxin B subunit induce antigen-specific Foxp3(+)regulatory T cells and protect against experimental autoimmune encephalomyelitis.J Immunol. 2012 Feb 15;188(4):1686-97. Epub 2012 Jan 16. PubMed PMID: 22250081.

173: Lee E, Chanamara S, Pleasure D, Soulika AM. IFN-gamma signaling in thecentral nervous system controls the course of experimental autoimmuneencephalomyelitis independently of the localization and composition of

inflammatory foci. J Neuroinflammation. 2012 Jan 16;9:7. PubMed PMID: 22248039;PubMed Central PMCID: PMC3293042.

174: Bodhankar S, Offner H. GPR30 FORMS AN INTEGRAL PART OF E2-PROTECTIVE PATHWAYIN EXPERIMENTAL AUTOIMMUNE ENCEPHALOMYELITIS. Immunol Endocr Metab Agents MedChem. 2011 Dec;11(4):262-274. PubMed PMID: 22247749; PubMed Central PMCID:PMC3255092.

175: Castro SB, Junior CO, Alves CC, Dias AT, Alves LL, Mazzoccoli L, MesquitaFP, Figueiredo NS, Juliano MA, Castañon MC, Gameiro J, Almeida MV, Teixeira HC,Ferreira AP. Immunomodulatory effects and improved prognosis of experimentalautoimmune encephalomyelitis after O-tetradecanoyl-genistein treatment. IntImmunopharmacol. 2012 Feb;12(2):465-70. Epub 2012 Jan 13. PubMed PMID: 22245971.

176: Cusick MF, Libbey JE, Trede NS, Eckels DD, Fujinami RS. Human T cellexpansion and experimental autoimmune encephalomyelitis inhibited by Lenaldekar, a small molecule discovered in a zebrafish screen. J Neuroimmunol. 2012Mar;244(1-2):35-44. Epub 2012 Jan 14. PubMed PMID: 22245285.

177: Brod SA, Bauer V, Hood Z. Oral ACTH (H.P. Acthar(®)Gel) inhibits IL-1 andIL-17 secretion in humans. Biomed Pharmacother. 2012 Feb;66(1):36-9. Epub 2011Dec 27. PubMed PMID: 22244960.

178: Wang X, Zhang Y, Yang XO, Nurieva RI, Chang SH, Ojeda SS, Kang HS, SchlunsKS, Gui J, Jetten AM, Dong C. Transcription of Il17 and Il17f is controlled byconserved noncoding sequence 2. Immunity. 2012 Jan 27;36(1):23-31. Epub 2012 Jan 12. PubMed PMID: 22244845; PubMed Central PMCID: PMC3270375.

179: Xin J, Feinstein DL, Hejna MJ, Lorens SA, McGuire SO. Beneficial Effects of Blueberries in Experimental Autoimmune Encephalomyelitis. J Agric Food Chem. 2012Feb 10. [Epub ahead of print] PubMed PMID: 22243431.

180: Yu CR, Lee YS, Mahdi RM, Surendran N, Egwuagu CE. Therapeutic targeting of STAT3 (signal transducers and activators of transcription 3) pathway inhibitsexperimental autoimmune uveitis. PLoS One. 2012;7(1):e29742. Epub 2012 Jan 5.PubMed PMID: 22238646; PubMed Central PMCID: PMC3252323.

181: Guo F, Maeda Y, Ko EM, Delgado M, Horiuchi M, Soulika A, Miers L, Burns T,Itoh T, Shen H, Lee E, Sohn J, Pleasure D. Disruption of NMDA receptors inoligodendroglial lineage cells does not alter their susceptibility to

experimental autoimmune encephalomyelitis or their normal development. JNeurosci. 2012 Jan 11;32(2):639-45. PubMed PMID: 22238099.

182: Nevin RL. Investigating channel blockers for the treatment of multiplesclerosis: considerations with mefloquine and carbenoxolone. J Neuroimmunol. 2012Feb 29;243(1-2):106-7. Epub 2012 Jan 10. PubMed PMID: 22236373.

183: Fagone P, Mangano K, Coco M, Perciavalle V, Garotta G, Romao CC, NicolettiF. Therapeutic potential of carbon monoxide in multiple sclerosis. Clin ExpImmunol. 2012 Feb;167(2):179-87. doi: 10.1111/j.1365-2249.2011.04491.x. Review.PubMed PMID: 22235993; PubMed Central PMCID: PMC3278683.

184: Jenh CH, Cox MA, Cui L, Reich EP, Sullivan L, Chen SC, Kinsley D, Qian S,Kim SH, Rosenblum S, Kozlowski J, Fine JS, Zavodny PJ, Lundell D. A selective andpotent CXCR3 antagonist SCH 546738 attenuates the development of autoimmunediseases and delays graft rejection. BMC Immunol. 2012 Jan 10;13(1):2. [Epubahead of print] PubMed PMID: 22233170; PubMed Central PMCID: PMC3298469.

185: Pöllinger B. IL-17 producing T cells in mouse models of multiple sclerosisand rheumatoid arthritis. J Mol Med (Berl). 2012 Jun;90(6):613-24. Epub 2012 Jan 10. PubMed PMID: 22231742.

186: Huang G, Wang Y, Vogel P, Kanneganti TD, Otsu K, Chi H. Signaling via thekinase p38α programs dendritic cells to drive TH17 differentiation and autoimmuneinflammation. Nat Immunol. 2012 Jan 8;13(2):152-61. doi: 10.1038/ni.2207. PubMed PMID: 22231518; PubMed Central PMCID: PMC3262925.

187: Axtell RC, Raman C, Steinman L. Type I Interferons: Beneficial in Th1 andDetrimental in Th17 Autoimmunity. Clin Rev Allergy Immunol. 2012 Jan 10. [Epub ahead of print] PubMed PMID: 22231516.

188: Murakami H, Wang Y, Hasuwa H, Maeda Y, Kinoshita T, Murakami Y. Enhanced response of T lymphocytes from Pgap3 knockout mouse: Insight into roles of fatty acid remodeling of GPI anchored proteins. Biochem Biophys Res Commun. 2012 Jan 27;417(4):1235-41. Epub 2011 Dec 29. PubMed PMID: 22227195.

189: Zaheer S, Wu Y, Yang X, Ahrens M, Sahu SK, Zaheer A. Clinical course of myelin oligodendrocyte glycoprotein 35-55 induced experimental autoimmune encephalomyelitis is aggravated by glia maturation factor. Neurochem Int. 2012 Feb;60(3):215-9. Epub 2011 Dec 30. PubMed PMID: 22226840; PubMed Central PMCID:PMC3288541.

190: Ramaglia V, Hughes TR, Donev RM, Ruseva MM, Wu X, Huitinga I, Baas F, NealJW, Morgan BP. C3-dependent mechanism of microglial priming relevant to multiple sclerosis. Proc Natl Acad Sci U S A. 2012 Jan 17;109(3):965-70. Epub 2012 Jan 4. PubMed PMID: 22219359; PubMed Central PMCID: PMC3271873

191: Kitazawa Y, Warabi Y, Bandoh M, Takahashi T, Matsubara S. Elderly-onsetneuromyelitis optica which developed after the diagnosis of prostateadenocarcinoma and relapsed after a 23-valent pneumococcal polysaccharide

vaccination. Intern Med. 2012;51(1):103-7. Epub 2012 Jan 1. PubMed PMID:22214633.

192: Ioannou M, Alissafi T, Lazaridis I, Deraos G, Matsoukas J, Gravanis A,Mastorodemos V, Plaitakis A, Sharpe A, Boumpas D, Verginis P. Crucial role ofgranulocytic myeloid-derived suppressor cells in the regulation of central

nervous system autoimmune disease. J Immunol. 2012 Feb 1;188(3):1136-46. Epub2011 Dec 30. PubMed PMID: 22210912

193: Badawi AH, Kiptoo P, Wang WT, Choi IY, Lee P, Vines CM, Siahaan TJ.Suppression of EAE and prevention of blood-brain barrier breakdown aftervaccination with novel bifunctional peptide inhibitor. Neuropharmacology. 2012Mar;62(4):1874-81. Epub 2011 Dec 17. PubMed PMID: 22210333; PubMed Central PMCID:PMC3269550.

194: Spence RD, Voskuhl RR. Neuroprotective effects of estrogens and androgens inCNS inflammation and neurodegeneration. Front Neuroendocrinol. 2012Jan;33(1):105-15. Epub 2011 Dec 24.. REVIEW

195: Buenafe AC. Diurnal rhythms are altered in a mouse model of multiplesclerosis. J Neuroimmunol. 2012 Feb 29;243(1-2):12-7. Epub 2011 Dec 30. PubMedPMID: 22209286.USA,

196: Abbott DJ, Blanchfield JL, Martinson DA, Russell SC, Taslim N, Curtis AD,Mannie MD. Neuroantigen-specific, tolerogenic vaccines: GM-CSF is a fusionpartner that facilitates tolerance rather than immunity to dominant self-epitopesof myelin in murine models of experimental autoimmune encephalomyelitis (EAE).BMC Immunol. 2011 Dec 30;12:72. PubMed PMID: 22208499; PubMed Central PMCID:PMC3261124

197: Mader S, Gredler V, Schanda K, Rostasy K, Dujmovic I, Pfaller K, Lutterotti A, Jarius S, Di Pauli F, Kuenz B, Ehling R, Hegen H, Deisenhammer F, Aboul-Enein F, Storch MK, Koson P, Drulovic J, Kristoferitsch W, Berger T, Reindl M.Complement activating antibodies to myelin oligodendrocyte glycoprotein in neuromyelitis optica and related disorders. J Neuroinflammation. 2011 Dec 28;8:184. PubMed PMID: 22204662; PubMed Central PMCID: PMC3278385. NOT EAE

198: Chen SJ, Wang YL, Fan HC, Lo WT, Wang CC, Sytwu HK. Current status of the immunomodulation and immunomediated therapeutic strategies for multiplesclerosis. Clin Dev Immunol. 2012;2012:970789. Epub 2011 Dec 6. Review. PubMedPMID: 22203863; PubMed Central PMCID: PMC3235500.REVIEW

199: Wuerfel E, Smyth M, Millward JM, Schellenberger E, Glumm J, Prozorovski T, Aktas O, Schulze-Topphoff U, Schnorr J, Wagner S, Taupitz M, Infante-Duarte C,

Wuerfel J. Electrostatically Stabilized Magnetic Nanoparticles - An Optimized Protocol to Label Murine T Cells for in vivo MRI. Front Neurol. 2011;2:72. Epub 2011 Dec 16

200: Lucchese G, Sinha AA, Kanduc D. How a single amino acid change may alter the immunological information of a peptide. Front Biosci (Elite Ed). 2012 Jan 1;4:1843-52. PubMed PMID: 22202001

201: Son CG. Safety of 4-week indirect-moxibustion therapy at CV4 and CV8. J Acupunct Meridian Stud. 2011 Dec;4(4):262-5. doi: 10.1016/j.jams.2011.09.018.Epub 2011 Oct 19. PubMed PMID: 22196510. NOT EAE

202: Salem M, Mony JT, Løbner M, Khorooshi R, Owens T. Interferon regulatory factor-7 modulates experimental autoimmune encephalomyelitis in mice. J Neuroinflammation. 2011 Dec 23;8:181. PubMed PMID: 22196084; PubMed Central PMCID: PMC3260126.

203: Kovalchin J, Krieger J, Genova M, Kawamoto N, Augustyniak M, Collins K, Bloom T, Masci A, Hittinger T, Dufour I, Strominger JL, Zanelli E Macrophage-specific chemokines induced via innate immunity by amino acid

copolymers and their role in EAE. PLoS One. 2011;6(12):e26274. Epub 2011 Dec 15.

204: Oksenberg JR, Hauser SL. Decoding multiple sclerosis. Ann Neurol. 2011 Dec;70(6):A5-7. doi: 10.1002/ana.22680. PubMed PMID: 22190375.REVIEW

205: Moreno B, Jukes JP, Vergara-Irigaray N, Errea O, Villoslada P, Perry VH, Newman TA. Systemic inflammation induces axon injury during brain inflammation. Ann Neurol. 2011 Dec;70(6):932-42. doi: 10.1002/ana.22550. PubMed PMID: 22190366

206: Blankenhorn EP, Butterfield R, Case LK, Wall EH, del Rio R, Diehl SA, Krementsov DN, Saligrama N, Teuscher C. Genetics of experimental allergic encephalomyelitis supports the role of T helper cells in multiple sclerosis

pathogenesis. Ann Neurol. 2011 Dec;70(6):887-96.

207: Jain MR, Li Q, Liu T, Rinaggio J, Ketkar A, Tournier V, Madura K, Elkabes S, Li H. Proteomic identification of immunoproteasome accumulation in formalin-fixed rodent spinal cords with experimental autoimmune encephalomyelitis. J Proteome Res. 2012 Mar 2;11(3):1791-803. Epub 2012 Feb 6. PubMed PMID: 22188123;

208: Kubo T, Tokita S, Yamashita T. Repulsive Guidance Molecule-a and Demyelination: Implications for Multiple Sclerosis. J Neuroimmune Pharmacol. 2011 Dec 21. [Epub ahead of print] PubMed PMID: 22183806.

209: MacKenzie-Graham A, Rinek GA, Avedisian A, Gold SM, Frew AJ, Aguilar C, Lin DR, Umeda E, Voskuhl RR, Alger JR. Cortical atrophy in experimental autoimmune encephalomyelitis: in vivo imaging. Neuroimage. 2012 Mar;60(1):95-104. Epub 2011 Dec 13. PubMed PMID: 22182769; PubMed Central PMCID: PMC3293104.

210: Decressac M, Mattsson B, Lundblad M, Weikop P, Björklund A. Progressive neurodegenerative and behavioural changes induced by AAV-mediated overexpression of α-synuclein in midbrain dopamine neurons. Neurobiol Dis. 2012 Mar;45(3):939-53. Epub 2011 Dec 11. PubMed PMID: 22182688.

211: Peters A, Pitcher LA, Sullivan JM, Mitsdoerffer M, Acton SE, Franz B, Wucherpfennig K, Turley S, Carroll MC, Sobel RA, Bettelli E, Kuchroo VK. Th17 cells induce ectopic lymphoid follicles in central nervous system tissue

inflammation. Immunity. 2011 Dec 23;35(6):986-96. Epub 2011 Dec 15. PubMed PMID: 22177922.

212: Zhang X, Li QY, Xiao BG. Anti-inflammatory effect of erythropoietin therapy on experimental autoimmune encephalomyelitis. Int J Neurosci. 2012 May;122(5):255-62. Epub 2012 Feb 6. PubMed PMID: 22176555.

213: Adamus G, Brown L, Andrew S, Meza-Romero R, Burrows GG, Vandenbark AA. Neuroprotective effects of recombinant T-cell receptor ligand in autoimmune optic neuritis in HLA-DR2 mice. Invest Ophthalmol Vis Sci. 2012 Jan 25;53(1):406-12. Print 2012 Jan. PubMed PMID: 22167100; PubMed Central PMCID: PMC3292374.

214: Zhang L, Yuan S, Cheng G, Guo B. Type I IFN promotes IL-10 production from T cells to suppress Th17 cells and Th17-associated autoimmune inflammation. PLoS One. 2011;6(12):e28432. Epub 2011 Dec 6.

215: Tang X, Tian L, Esteso G, Choi SC, Barrow AD, Colonna M, Borrego F, Coligan JE. Leukocyte-associated Ig-like receptor-1-deficient mice have an altered immune cell phenotype. J Immunol. 2012 Jan 15;188(2):548-58. Epub 2011 Dec 7. PubMedPMID: 22156345; PubMed Central PMCID: PMC3286132.

216: Dutra RC, de Souza PR, Bento AF, Marcon R, Bicca MA, Pianowski LF, Calixto JB. Euphol prevents experimental autoimmune encephalomyelitis in mice: evidence for the underlying mechanisms. Biochem Pharmacol. 2012 Feb 15;83(4):531-42. Epub 2011 Dec 1. PubMed PMID: 22155310.

217: Thöne J, Ellrichmann G, Seubert S, Peruga I, Lee DH, Conrad R, Hayardeny L, Comi G, Wiese S, Linker RA, Gold R. Modulation of autoimmune demyelination bylaquinimod via induction of brain-derived neurotrophic factor. Am J Pathol. 2012 Jan;180(1):267-74. Epub 2011 Dec 5. PubMed PMID: 22152994.

218: del Rio R, Noubade R, Saligrama N, Wall EH, Krementsov DN, Poynter ME,Zachary JF, Thurmond RL, Teuscher C. Histamine H4 receptor optimizes T regulatorycell frequency and facilitates anti-inflammatory responses within the centralnervous system. J Immunol. 2012 Jan 15;188(2):541-7. Epub 2011 Dec 5. PubMedPMID: 22147765; PubMed Central PMCID: PMC3253209.

219: Cruz-Orengo L, Chen YJ, Kim JH, Dorsey D, Song SK, Klein RS. CXCR7antagonism prevents axonal injury during experimental autoimmuneencephalomyelitis as revealed by in vivo axial diffusivity. J Neuroinflammation.

2011 Dec 6;8:170. PubMed PMID: 22145790; PubMed Central PMCID: PMC3305694.

220: Alvarez JI, Dodelet-Devillers A, Kebir H, Ifergan I, Fabre PJ, Terouz S,Sabbagh M, Wosik K, Bourbonnière L, Bernard M, van Horssen J, de Vries HE,Charron F, Prat A. The Hedgehog pathway promotes blood-brain barrier integrityand CNS immune quiescence. Science. 2011 Dec 23;334(6063):1727-31. Epub 2011 Dec 1. PubMed PMID: 22144466.

221: Dann A, Poeck H, Croxford AL, Gaupp S, Kierdorf K, Knust M, Pfeifer D,Maihoefer C, Endres S, Kalinke U, Meuth SG, Wiendl H, Knobeloch KP, Akira S,Waisman A, Hartmann G, Prinz M. Cytosolic RIG-I-like helicases act as negativeregulators of sterile inflammation in the CNS. Nat Neurosci. 2011 Dec4;15(1):98-106. doi: 10.1038/nn.2964. PubMed PMID: 22138643

222: Scerbo MJ, Bibolini MJ, Roth GA, Monferran CG. Synapsin peptide fused to E. coli heat-labile toxin B subunit induces regulatory T cells and modulatescytokine balance in experimental autoimmune encephalomyelitis. J Neuroimmunol.2012 Jan 18;242(1-2):16-25. Epub 2011 Dec 3. PubMed PMID: 22138356.

223: Owens GP, Gilden D, Burgoon MP, Yu X, Bennett JL. Viruses and multiplesclerosis. Neuroscientist. 2011 Dec;17(6):659-76. Review. PubMed PMID: 22130640; PubMed Central PMCID: PMC3293404.

224: Menon R, Di Dario M, Cordiglieri C, Musio S, La Mantia L, Milanese C, DiStefano AL, Crabbio M, Franciotta D, Bergamaschi R, Pedotti R, Medico E, FarinaC. Gender-based blood transcriptomes and interactomes in multiple sclerosis:involvement of SP1 dependent gene transcription. J Autoimmun. 2012May;38(2-3):J144-55. Epub 2011 Nov 25. PubMed PMID: 22119415.

225: Baker D, Amor S. Publication guidelines for refereeing and reporting onanimal use in experimental autoimmune encephalomyelitis. J Neuroimmunol. 2012 Jan18;242(1-2):78-83. Epub 2011 Nov 26. PubMed PMID: 22119102.

226: Joseph J, Bittner S, Kaiser FM, Wiendl H, Kissler S. IL-17 silencing doesnot protect nonobese diabetic mice from autoimmune diabetes. J Immunol. 2012 Jan 1;188(1):216-21. Epub 2011 Nov 23. PubMed PMID: 22116823.

227: Tsakiri N, Papadopoulos D, Denis MC, Mitsikostas DD, Kollias G. TNFR2 onnon-haematopoietic cells is required for Foxp3+ Treg-cell function and diseasesuppression in EAE. Eur J Immunol. 2012 Feb;42(2):403-12. doi:

10.1002/eji.201141659. Epub 2011 Dec 16. PubMed PMID: 22105853.

228: Kong S, McBurney MW, Fang D. Sirtuin 1 in immune regulation andautoimmunity. Immunol Cell Biol. 2012 Jan;90(1):6-13. doi: 10.1038/icb.2011.102. Epub 2011 Nov 22. Review. PubMed PMID: 22105513.

229: Dai H, Ciric B, Zhang GX, Rostami A. Bowman-Birk Inhibitor attenuatesexperimental autoimmune encephalomyelitis by delaying infiltration ofinflammatory cells into the CNS. Immunol Res. 2011 Dec;51(2-3):145-52. PubMedPMID: 22095543.

230: Sternberg Z, Cesario A, Rittenhouse-Olson K, Sobel RA, Leung YK, PankewyczO, Zhu B, Whitcomb T, Sternberg DS, Munschauer FE. Acamprosate modulatesexperimental autoimmune encephalomyelitis. Inflammopharmacology. 2012Feb;20(1):39-48. Epub 2011 Nov 17. Erratum in: Inflammopharmacology. 2012Feb;20(1):49. Leung, Yi-Kan [added]. PubMed PMID: 22090150.

231: Guerau-de-Arellano M, Smith KM, Godlewski J, Liu Y, Winger R, Lawler SE,Whitacre CC, Racke MK, Lovett-Racke AE. Micro-RNA dysregulation in multiplesclerosis favours pro-inflammatory T-cell-mediated autoimmunity. Brain. 2011Dec;134(Pt 12):3578-89. Epub 2011 Nov 15. PubMed PMID: 22088562; PubMed CentralPMCID: PMC3235556

232: Batoulis H, Recks MS, Addicks K, Kuerten S. Experimental autoimmuneencephalomyelitis--achievements and prospective advances. APMIS. 2011Dec;119(12):819-30. doi: 10.1111/j.1600-0463.2011.02794.x. Epub 2011 Oct 18.Review. PubMed PMID: 22085358.

233: Glatigny S, Duhen R, Oukka M, Bettelli E. Cutting edge: loss of α4 integrin expression differentially affects the homing of Th1 and Th17 cells. J Immunol.2011 Dec 15;187(12):6176-9. Epub 2011 Nov 14. PubMed PMID: 22084440; PubMedCentral PMCID: PMC3237912.

234: Wellejus A, Elbrønd-Bek H, Kelly NM, Weidner MS, Jørgensen SH. 4-iodophenyl isothiocyanate: a neuroprotective compound. Restor Neurol Neurosci.2012;30(1):21-38. PubMed PMID: 22082767.

235: Macmillan CJ, Starkey RJ, Easton AS. Angiogenesis is regulated byangiopoietins during experimental autoimmune encephalomyelitis and is indirectly related to vascular permeability. J Neuropathol Exp Neurol. 2011

Dec;70(12):1107-23. PubMed PMID: 22082662.

236: Ruggiero V. Involvement of IL-1R/TLR signalling in experimental autoimmune encephalomyelitis and multiple sclerosis. Curr Mol Med. 2012 Mar 1;12(3):218-36. PubMed PMID: 22082484.

237: Loschko J, Heink S, Hackl D, Dudziak D, Reindl W, Korn T, Krug AB. Antigentargeting to plasmacytoid dendritic cells via Siglec-H inhibits Th cell-dependentautoimmunity. J Immunol. 2011 Dec 15;187(12):6346-56. Epub 2011 Nov 11. PubMedPMID: 22079988.

238: Kruglov AA, Lampropoulou V, Fillatreau S, Nedospasov SA. Pathogenic andprotective functions of TNF in neuroinflammation are defined by its expression inT lymphocytes and myeloid cells. J Immunol. 2011 Dec 1;187(11):5660-70. Epub 2011Nov 4. PubMed PMID: 22058414.

239: Massilamany C, Asojo OA, Gangaplara A, Steffen D, Reddy J. Identification ofa second mimicry epitope from Acanthamoeba castellanii that induces CNSautoimmunity by generating cross-reactive T cells for MBP 89-101 in SJL mice. IntImmunol. 2011 Dec;23(12):729-39. Epub 2011 Nov 4. PubMed PMID: 22058327.

240: Ifergan I, Kebir H, Alvarez JI, Marceau G, Bernard M, Bourbonnière L,Poirier J, Duquette P, Talbot PJ, Arbour N, Prat A. Central nervous systemrecruitment of effector memory CD8+ T lymphocytes during neuroinflammation isdependent on α4 integrin. Brain. 2011 Dec;134(Pt 12):3560-77. Epub 2011 Nov 4.PubMed PMID: 22058139.

241: Tepavčević V, Lazarini F, Alfaro-Cervello C, Kerninon C, Yoshikawa K,Garcia-Verdugo JM, Lledo PM, Nait-Oumesmar B, Baron-Van Evercooren A.Inflammation-induced subventricular zone dysfunction leads to olfactory deficits in a targeted mouse model of multiple sclerosis. J Clin Invest. 2011Dec;121(12):4722-34. doi: 10.1172/JCI59145. Epub 2011 Nov 7. PubMed PMID:22056384; PubMed Central PMCID: PMC3226002.

242: Wang J, Ren Z, Xu Y, Xiao S, Meydani SN, Wu D. Epigallocatechin-3-gallateameliorates experimental autoimmune encephalomyelitis by altering balance amongCD4+ T-cell subsets. Am J Pathol. 2012 Jan;180(1):221-34. Epub 2011 Nov 3. PubMedPMID: 22056360.

243: Zheng XP, Zhang HL, Li HF, Zhang MZ, Qiu W, Hu XQ. Neuroprotective potentialbeyond immunoregulation of helminth infection as a therapeutic target in multiplesclerosis. Med Hypotheses. 2012 Jan;78(1):95-7. Epub 2011 Nov 1. PubMed PMID:22047987.

244: Banquy X, Kristiansen K, Lee DW, Israelachvili JN. Adhesion and hemifusionof cytoplasmic myelin lipid membranes are highly dependent on the lipidcomposition. Biochim Biophys Acta. 2012 Mar;1818(3):402-10. Epub 2011 Oct 25.PubMed PMID: 22047743; PubMed Central PMCID: PMC3273667.

245: Buenafe AC, Andrew S, Offner H, Vandenbark AA. Regulatory T cells play arole in T-cell receptor CDR2 peptide regulation of experimental autoimmuneencephalomyelitis. Immunology. 2012 Feb;135(2):168-79. doi:

10.1111/j.1365-2567.2011.03531.x. PubMed PMID: 22044096; PubMed Central PMCID:PMC3277719.

246: Xie L, Yamasaki T, Ichimaru N, Yui J, Kawamura K, Kumata K, Hatori A,Nonomura N, Zhang MR, Li XK, Takahara S. [(11)C]DAC-PET for noninvasivelymonitoring neuroinflammation and immunosuppressive therapy efficacy in ratexperimental autoimmune encephalomyelitis model. J Neuroimmune Pharmacol. 2012Mar;7(1):231-42. Epub 2011 Oct 29. PubMed PMID: 22038158.

247: Endong L, Shijie J, Sonobe Y, Di M, Hua L, Kawanokuchi J, Mizuno T, SuzumuraA. The gap-junction inhibitor carbenoxolone suppresses the differentiation ofTh17 cells through inhibition of IL-23 expression in antigen presenting cells. J Neuroimmunol. 2011 Dec 15;240-241:58-64. Epub 2011 Oct 28. PubMed PMID: 22036952.

248: Chanaday NL, de Bem AF, Roth GA. Effect of diphenyl diselenide on thedevelopment of experimental autoimmune encephalomyelitis. Neurochem Int. 2011Dec;59(8):1155-62. Epub 2011 Oct 19. PubMed PMID: 22032971.

249: Gonzalez-Cabrera PJ, Cahalan SM, Nguyen N, Sarkisyan G, Leaf NB, Cameron MD,Kago T, Rosen H. S1P(1) receptor modulation with cyclical recovery fromlymphopenia ameliorates mouse model of multiple sclerosis. Mol Pharmacol. 2012Feb;81(2):166-74. Epub 2011 Oct 26. PubMed PMID: 22031473; PubMed Central PMCID: PMC3263953.

250: Alli R, Nguyen P, Geiger TL. Altered differentiation, diminishedpathogenicity, and regulatory activity of myelin-specific T cells expressing anenhanced affinity TCR. J Immunol. 2011 Dec 1;187(11):5521-31. Epub 2011 Oct 24.

PubMed PMID: 22025553; PubMed Central PMCID: PMC3221875.

251: Lou ZY, Chen C, He Q, Zhao CB, Xiao BG. Targeting CB(2) receptor as aneuroinflammatory modulator in experimental autoimmune encephalomyelitis. MolImmunol. 2011 Dec;49(3):453-61. Epub 2011 Oct 22. PubMed PMID: 22024414.

252: Shimamura M, Sato N, Morishita R. Experimental and clinical application ofplasmid DNA in the field of central nervous diseases. Curr Gene Ther. 2011Dec;11(6):491-500. PubMed PMID: 22023479.

253: Wu D, Wang J, Pae M, Meydani SN. Green tea EGCG, T cells, and Tcell-mediated autoimmune diseases. Mol Aspects Med. 2012 Feb;33(1):107-18. Epub2011 Oct 14. Review. PubMed PMID: 22020144.

254: Huarte E, Rynda-Apple A, Riccardi C, Skyberg JA, Golden S, Rollins MF,Ramstead AG, Jackiw LO, Maddaloni M, Pascual DW. Tolerogen-inducedinterferon-producing killer dendritic cells (IKDCs) protect against EAE. J

Autoimmun. 2011 Dec;37(4):328-41. Epub 2011 Oct 22. PubMed PMID: 22018711; PubMedCentral PMCID: PMC3237120.

255: Jagessar SA ,Gran B, Heijmans N, Bauer J, Laman JD, 't Hart BA, Constantinescu CS.Discrepant effects of human interferon-gamma on clinical and immunological disease parameters in a novel marmoset model for multiple sclerosis. J Neuroimmune Pharmacol. 2012 Mar;7(1):253-65. Epub 2011 Oct 20. PMID:22012268[PubMed - indexed for MEDLINE] PMCID:PMC3280389

256: Lee DH, Geyer E, Flach AC, Jung K, Gold R, Flügel A, Linker RA, Lühder F.Central nervous system rather than immune cell-derived BDNF mediates axonalprotective effects early in autoimmune demyelination. Acta Neuropathol. 2012Feb;123(2):247-58. Epub 2011 Oct 19. PubMed PMID: 22009304; PubMed Central PMCID:PMC3259380.

257: Williams R, Buchheit CL, Berman NE, LeVine SM. Pathogenic implications ofiron accumulation in multiple sclerosis. J Neurochem. 2012 Jan;120(1):7-25. doi: 10.1111/j.1471-4159.2011.07536.x. Epub 2011 Nov 11. Review. Erratum in: JNeurochem. 2012 Apr;121(2):326. PubMed PMID: 22004421; PubMed Central PMCID:PMC3295237.

258: Cantorna MT, Zhao J, Yang L. Vitamin D, invariant natural killer T-cells andexperimental autoimmune disease. Proc Nutr Soc. 2012 Feb;71(1):62-6. PubMed PMID21996367.

259: Subramanian S, Miller LM, Grafe MR, Vandenbark AA, Offner H. Contribution ofGPR30 for 1,25 dihydroxyvitamin D₃ protection in EAE. Metab Brain Dis. 2012Mar;27(1):29-35. Epub 2011 Oct 13. PubMed PMID: 21994003.

260: Lalive PH, Molnarfi N, Benkhoucha M, Weber MS, Santiago-Raber ML. Antibodyresponse in MOG(35-55) induced EAE. J Neuroimmunol. 2011 Dec 15;240-241:28-33.Epub 2011 Oct 10. PubMed PMID: 21993076.

261: Ren X, Akiyoshi K, Grafe MR, Vandenbark AA, Hurn PD, Herson PS, Offner H.Myelin specific cells infiltrate MCAO lesions and exacerbate stroke severity.Metab Brain Dis. 2012 Mar;27(1):7-15. Epub 2011 Oct 12. PubMed PMID: 21989743;

262: Liu G, Muili KA, Agashe VV, Lyons JA. Unique B cell responses in Bcell-dependent and B cell-independent EAE. Autoimmunity. 2012 May;45(3):199-209. Epub 2011 Oct 11. PubMed PMID: 21985327.

263: Srinivasan M, Janardhanam S. Novel p65 binding glucocorticoid-inducedleucine zipper peptide suppresses experimental autoimmune encephalomyelitis. JBiol Chem. 2011 Dec 30;286(52):44799-810. Epub 2011 Sep 29. PubMed PMID:21965677; PubMed Central PMCID: PMC3247947.

264: Bibolini MJ, Chanaday NL, Báez NS, Degano AL, Monferran CG, Roth GA.Inhibitory role of diazepam on autoimmune inflammation in rats with experimental autoimmune encephalomyelitis. Neuroscience. 2011 Dec 29;199:421-8. Epub 2011 Sep 17. PubMed PMID: 21964471.

265: Kiraly A, Koffman B, Hacker M, Gunning W, Rasche S, Quinn A. A novelaza-anthrapyrazole blocks the progression of experimental autoimmuneencephalomyelitis after the priming of autoimmunity. Clin Immunol. 2011

Dec;141(3):304-16. Epub 2011 Aug 30. PubMed PMID: 21964417.

266: Piconese S, Costanza M, Tripodo C, Sangaletti S, Musio S, Pittoni P, PolianiPL, Burocchi A, Passafaro AL, Gorzanelli A, Vitali C, Chiodoni C, Barnaba V,Pedotti R, Colombo MP. The matricellular protein SPARC supports folliculardendritic cell networking toward Th17 responses. J Autoimmun. 2011Dec;37(4):300-10. Epub 2011 Sep 29. PubMed PMID: 21962567.

267: Sajic M, Gregson N, Linington C, Hughes RA, Smith KJ. The role of CD8(+) Tcells in a model of multiple sclerosis induced with recombinant myelinoligodendrocyte glycoprotein. Mult Scler. 2012 Mar;18(3):286-98. Epub 2011 Sep27. PubMed PMID: 21952095.

268: Wu L, Zepp J, Li X. Function of Act1 in IL-17 family signaling and autoimmunity. Adv Exp Med Biol. 2012;946:223-35. Review. PubMed PMID: 21948371.

269: Mueggler T, Pohl H, Baltes C, Riethmacher D, Suter U, Rudin M. MRI signaturein a novel mouse model of genetically induced adult oligodendrocyte cell death.Neuroimage. 2012 Jan 16;59(2):1028-36. Epub 2011 Sep 10. PubMed PMID: 21945466.

270: Kuerten S, Lehmann PV. The immune pathogenesis of experimental autoimmuneencephalomyelitis: lessons learned for multiple sclerosis? J Interferon Cytokine Res. 2011 Dec;31(12):907-16. Epub 2011 Sep 21. Review. PubMed PMID: 21936633.

271: Fernandes de Abreu DA, Landel V, Féron F. Seasonal, gestational andpostnatal influences on multiple sclerosis: the beneficial role of a vitamin Dsupplementation during early life. J Neurol Sci. 2011 Dec 15;311(1-2):64-8. Epub 2011 Sep 17. PubMed PMID: 21930286.

272: Pletinckx K, Stijlemans B, Pavlovic V, Laube R, Brandl C, Kneitz S, Beschin A, De Baetselier P, Lutz MB. Similar inflammatory DC maturation signaturesinduced by TNF or Trypanosoma brucei antigens instruct default Th2-cell

responses. Eur J Immunol. 2011 Dec;41(12):3479-94. doi: 10.1002/eji.201141631.Epub 2011 Nov 3. PubMed PMID: 21928284.

273: Jagessar SA, Heijmans N, Blezer EL, Bauer J, Blokhuis JH, Wubben JA,Drijfhout JW, van den Elsen PJ, Laman JD, Hart BA. Unravelling theT-cell-mediated autoimmune attack on CNS myelin in a new primate EAE model

induced with MOG34-56 peptide in incomplete adjuvant. Eur J Immunol. 2012Jan;42(1):217-27. doi: 10.1002/eji.201141863. Epub 2011 Dec 2. PubMed PMID:21928277.

274: Cassani B, Villablanca EJ, Quintana FJ, Love PE, Lacy-Hulbert A, Blaner WS, Sparwasser T, Snapper SB, Weiner HL, Mora JR. Gut-tropic T cells that expressintegrin α4β7 and CCR9 are required for induction of oral immune tolerance inmice. Gastroenterology. 2011 Dec;141(6):2109-18. Epub 2011 Sep 16. PubMed PMID:21925467; PubMed Central PMCID: PMC3222333.

275: Lou ZY, Zhao CB, Xiao BG. Immunoregulation of experimental autoimmuneencephalomyelitis by the selective CB1 receptor antagonist. J Neurosci Res. 2012 Jan;90(1):84-95. doi: 10.1002/jnr.22721. Epub 2011 Sep 15. PubMed PMID: 21922514.

276: Nicolò C, Di Sante G, Migliara G, Valentini MG, Piermattei A, Delogu G, Ria F. Intracellular bacteria can cause EAE in SJL mice or modify self-specific Tcell repertoire. J Neurol Sci. 2011 Dec 15;311(1-2):103-6. Epub 2011 Sep 13.PubMed PMID: 21917274.

277: Boretius S, Escher A, Dallenga T, Wrzos C, Tammer R, Brück W, Nessler S,Frahm J, Stadelmann C. Assessment of lesion pathology in a new animal model of MSby multiparametric MRI and DTI. Neuroimage. 2012 Feb 1;59(3):2678-88. Epub 2011Sep 2. PubMed PMID: 21914485.

278: Serres S, Mardiguian S, Campbell SJ, McAteer MA, Akhtar A, Krapitchev A,Choudhury RP, Anthony DC, Sibson NR. VCAM-1-targeted magnetic resonance imagingreveals subclinical disease in a mouse model of multiple sclerosis. FASEB J. 2011Dec;25(12):4415-22. Epub 2011 Sep 9. PubMed PMID: 21908714.

279: Becher B, Segal BM. T(H)17 cytokines in autoimmune neuro-inflammation. Curr Opin Immunol. 2011 Dec;23(6):707-12. Epub 2011 Sep 9. Review. PubMed PMID:21907555.

280: Op De Beéck K, Maes L, Van den Bergh K, Derua R, Waelkens E, Van Steen K,Vermeersch P, Westhovens R, De Vlam K, Verschueren P, Hooijkaas H, Blockmans D,Bossuyt X. Heterogeneous nuclear RNPs as targets of autoantibodies in systemicrheumatic diseases. Arthritis Rheum. 2012 Jan;64(1):213-21. doi:10.1002/art.33327. PubMed PMID: 21905010.

281: Lassmann H, van Horssen J. The molecular basis of neurodegeneration inmultiple sclerosis. FEBS Lett. 2011 Dec 1;585(23):3715-23. Epub 2011 Aug 16.Review. PubMed PMID: 21854776.

282: Crespo O, Kang SC, Daneman R, Lindstrom TM, Ho PP, Sobel RA, Steinman L,Robinson WH. Tyrosine kinase inhibitors ameliorate autoimmune encephalomyelitisin a mouse model of multiple sclerosis. J Clin Immunol. 2011 Dec;31(6):1010-20.Epub 2011 Aug 17. PubMed PMID: 21847523; PubMed Central PMCID: PMC3225802.

283: Kollaee A, Ghaffarpor M, Pourmahmoudian H, Shahbazi M, Zamani M.Investigation of CD24 and Its expression in iranian relapsing-remitting multiple sclerosis. Int J Neurosci. 2011 Dec;121(12):684-90. Epub 2011 Sep 12. PubMedPMID: 21815873.

284: Gillingwater TH. Targeting synaptic pathology in multiple sclerosisfingolimod to the rescue? Br J Pharmacol. 2012 Feb;165(4):858-60. doi:10.1111/j.1476-5381.2011.01612.x. PubMed PMID: 21806598; PubMed Central PMCID:PMC3312483.

285: Biton A, Ansorge S, Bank U, Täger M, Reinhold D, Brocke S. Divergent actionsby inhibitors of DP IV and APN family enzymes on CD4+ Teff cell motility andfunctions. Immunobiology. 2011 Dec;216(12):1295-301. Epub 2011 Jul 7. PubMedPMID: 21802166.

286: Mowry EM. Vitamin D: evidence for its role as a prognostic factor inmultiple sclerosis. J Neurol Sci. 2011 Dec 15;311(1-2):19-22. Epub 2011 Jul 16.PubMed PMID: 21762931.

287: Rossi S, Lo Giudice T, De Chiara V, Musella A, Studer V, Motta C, BernardiG, Martino G, Furlan R, Martorana A, Centonze D. Oral fingolimod rescues thefunctional deficits of synapses in experimental autoimmune encephalomyelitis. BrJ Pharmacol. 2012 Feb;1654):861-9. doi: 10.1111/j.1476-5381.2011.01579.x. PubMedPMID: 21740406; PubMed Central PMCID: PMC3312484.

288: Tang EH, Libby P, Vanhoutte PM, Xu A. Anti-inflammation therapy byactivation of prostaglandin EP4 receptor in cardiovascular and other inflammatorydiseases. J Cardiovasc Pharmacol. 2012 Feb;59(2):116-23. Review. PubMed PMID:21697732; PubMed Central PMCID: PMC3191244.

289: Ingwersen J, Aktas O, Kuery P, Kieseier B, Boyko A, Hartung HP. Fingolimodin multiple sclerosis: mechanisms of action and clinical efficacy. Clin Immunol. 2012 Jan;142(1):15-24. doi: 10.1016/j.clim.2011.10.008. Epub 2011 May 26. Review.PubMed PMID: 21669553.

290: Kovacs JR, Li C, Yang Q, Li G, Garcia IG, Ju S, Roodman DG, Windle JJ, Zhangthe cell death machinery. Cell Death Differ. 2012 Jan;19(1):144-52. doi:10.1038/cdd.2011.78. Epub 2011 Jun 10. PubMed PMID: 21660048; PubMed CentralPMCID: PMC3252822.

291: Ji Z, Ke ZJ, Geng JG. SAP suppresses the development of experimentalautoimmune encephalomyelitis in C57BL/6 mice. Immunol Cell Biol. 2012Apr;90(4):388-95. doi: 10.1038/icb.2011.51. Epub 2011 Jun 7. PubMed PMID:

21647172.

292: Thamilarasan M, Koczan D, Hecker M, Paap B, Zettl UK. MicroRNAs in multiple sclerosis and experimental autoimmune encephalomyelitis. Autoimmun Rev. 2012Jan;11(3):174-9. doi: 10.1016/j.autrev.2011.05.009. Epub 2011 May 18. Review.PubMed PMID: 21621006.

293: Schweingruber N, Reichardt SD, Lühder F, Reichardt HM. Mechanisms ofglucocorticoids in the control of neuroinflammation. J Neuroendocrinol. 2012Jan;24(1):174-82. doi: 10.1111/j.1365-2826.2011.02161.x. Review. PubMed PMID:21615563.

294: Ghazavi A, Mosayebi G. The mechanism of sesame oil in amelioratingexperimental autoimmune encephalomyelitis in C57BL/6 mice. Phytother Res. 2012Jan;26(1):34-8. doi: 10.1002/ptr.3515. Epub 2011 Apr 28. PubMed PMID: 21538630.

295: Ehling P, Bittner S, Budde T, Wiendl H, Meuth SG. Ion channels in autoimmuneneurodegeneration. FEBS Lett. 2011 Dec 1;585(23):3836-42. Epub 2011 Apr 14.Review. PubMed PMID: 21501610.

296: Petermann F, Korn T. Cytokines and effector T cell subsets causingautoimmune CNS disease. FEBS Lett. 2011 Dec 1;585(23):3747-57. Epub 2011 Apr 6.Review. PubMed PMID: 21477588.

297: Junker A. Pathophysiology of translational regulation by microRNAs inmultiple sclerosis. FEBS Lett. 2011 Dec 1;585(23):3738-46. Epub 2011 Mar 29.Review. PubMed PMID: 21453702.

298: Pahan K. Immunomodulation of experimental allergic encephalomyelitis bycinnamon metabolite sodium benzoate. Immunopharmacol Immunotoxicol. 2011Dec;33(4):586-93. Epub 2011 Mar 22. PubMed PMID: 21425926; PubMed Central PMCID: PMC3206174.

299: Okuno T, Nakatsuji Y, Kumanogoh A. The role of immune semaphorins inmultiple sclerosis. FEBS Lett. 2011 Dec 1;585(23):3829-35. Epub 2011 Mar 22.Review. PubMed PMID: 21420960.

300: Sánchez AJ, García-Merino A. Neuroprotective agents: cannabinoids. ClinImmunol. 2012 Jan;142(1):57-67. doi: 10.1016/j.clim.2011.02.010. Epub 2011 Mar21. Review. PubMed PMID: 21420365.

301: Gold R, Linker RA, Stangel M. Fumaric acid and its esters: an emergingtreatment for multiple sclerosis with antioxidative mechanism of action. ClinImmunol. 2012 Jan;142(1):44-8. doi: 10.1016/j.clim.2011.02.017. Epub 2011 Feb 26.Review. PubMed PMID: 21414846.

302: Aronovich R, Katzav A, Chapman J. The strategies used for treatment ofexperimental autoimmune neuritis (EAN): a beneficial effect of glatiramer acetateadministered intraperitoneally. Clin Rev Allergy Immunol. 2012 Apr;42(2):181-8.PubMed PMID: 21234710.

303: Fissolo N, Montalban X, Comabella M. DNA-based vaccines for multiplesclerosis: current status and future directions. Clin Immunol. 2012Jan;142(1):76-83. doi: 10.1016/j.clim.2010.11.011. Epub 2010 Dec 15. Review.

PubMed PMID: 21163708.

304: Kandagaddala LD, Kang MJ, Chung BC, Patterson TA, Kwon OS. Expression andactivation of matrix metalloproteinase-9 and NADPH oxidase in tissues and plasma of experimental autoimmune encephalomyelitis in mice. Exp Toxicol Pathol. 2012Jan;64(1-2):109-14. Epub 2010 Aug 31. PubMed PMID: 20810258.

Supplementary Table 1. **Search results for statistical analysis of EAE Data**. Results of a Pubmed search using the term ‘*experimental encephalomyelitis’* during a 6 month time period between 1 December 2011 and 31 May 2012.
