# Supplementary material for: Two Years Later: Journals Are Not Yet Enforcing the ARRIVE Guidelines on Reporting Standards for Pre-Clinical Animal Studies
Source: PLoS Biol. 2014 Jan 7;12(1):e1001756. doi: 10.1371/journal.pbio.1001756 (PMC3883646; doi:10.1371/journal.pbio.1001756)
Supplement: Table S2 — Search results for statistical analysis of EAE data in high-impact-factor journals. Results of a PubMed search using the term “experimental encephalomyelitis” during a time period between 1 January 2010 and 31 August 2012 in Nature journals, Cell, and Science. (DOC) [file pbio.1001756.s002.doc]

**Supplementary Table 2**

Huang G, Wang Y, Vogel P, Kanneganti TD, Otsu K, Chi H. Signaling via the kinase p38α programs dendritic cells to drive TH17 differentiation and autoimmune inflammation. Nat Immunol. 2012;13:152-61.

Chuang HC, Lan JL, Chen DY, Yang CY, Chen YM, Li JP, Huang CY, Liu PE, Wang X, Tan TH. The kinase GLK controls autoimmunity and NF-κB signaling by activating the kinase PKC-θ in T cells. Nat Immunol. 2011;12:1113-8.

Codarri L, Gyülvészi G, Tosevski V, Hesske L, Fontana A, Magnenat L, Suter T, Becher B.RORγt drives production of the cytokine GM-CSF in helper T cells, which is essential for the effector phase of autoimmune neuroinflammation. Nat Immunol. 2011;12:560-7.

El-Behi M, Ciric B, Dai H, Yan Y, Cullimore M, Safavi F, Zhang GX, Dittel BN, Rostami A.The encephalitogenicity of T(H)17 cells is dependent on IL-1- and IL-23-induced production of the cytokine GM-CSF. Nat Immunol. 2011 ;12:568-75.

Delgoffe GM, Pollizzi KN, Waickman AT, Heikamp E, Meyers DJ, Horton MR, Xiao B, Worley PF, Powell JD. The kinase mTOR regulates the differentiation of helper T cells through the selective activation of signaling by mTORC1 and mTORC2.Nat Immunol. 2011;12:295-303.

Apetoh L, Quintana FJ, Pot C, Joller N, Xiao S, Kumar D, Burns EJ, Sherr DH, Weiner HL, Kuchroo VK. The aryl hydrocarbon receptor interacts with c-Maf to promote the differentiation of type 1 regulatory T cells induced by IL-27.Nat Immunol. 2010;11(9):854-61.

Rangachari M, Zhu C, Sakuishi K, Xiao S, Karman J, Chen A, Angin M, Wakeham A, Greenfield EA, Sobel RA, Okada H, McKinnon PJ, Mak TW, Addo MM, Anderson AC, Kuchroo VK.Bat3 promotes T cell responses and autoimmunity by repressing Tim-3–mediated cell death and exhaustion.Nat Med. 2012;18:1394-400

Zhu S, Pan W, Song X, Liu Y, Shao X, Tang Y, Liang D, He D, Wang H, Liu W, Shi Y, Harley JB, Shen N, Qian Y. The microRNA miR-23b suppresses IL-17-associated autoimmune inflammation by targeting TAB2, TAB3 and IKK-α. Nat Med. 2012; 18:1077-86.

Muramatsu R, Takahashi C, Miyake S, Fujimura H, Mochizuki H, Yamashita T. Angiogenesis induced by CNS inflammation promotes neuronal remodeling through vessel-derived prostacyclin. Nat Med. 2012;18:1658-64.

Mi S, Lee X, Hu Y, Ji B, Shao Z, Yang W, Huang G, Walus L, Rhodes K, Gong BJ, Miller RH, Pepinsky RB. Death receptor 6 negatively regulates oligodendrocyte survival, maturation and myelination. Nat Med. 2011;17:816-21.

Muramatsu R, Kubo T, Mori M, Nakamura Y, Fujita Y, Akutsu T, Okuno T, Taniguchi J, Kumanogoh A, Yoshida M, Mochizuki H, Kuwabara S, Yamashita T.RGMa modulates T cell responses and is involved in autoimmune encephalomyelitis. Nat Med. 2011;17:488-94.

Ponomarev ED, Veremeyko T, Barteneva N, Krichevsky AM, Weiner HL.

MicroRNA-124 promotes microglia quiescence and suppresses EAE by deactivating macrophages via the C/EBP-α-PU.1 pathway. Nat Med. 2011;17:64-70.

Fallarino F, Volpi C, Fazio F, Notartomaso S, Vacca C, Busceti C, Bicciato S, Battaglia G, Bruno V, Puccetti P, Fioretti MC, Nicoletti F, Grohmann U, Di Marco R.Metabotropic glutamate receptor-4 modulates adaptive immunity and restrains neuroinflammation.Nat Med. 2010;16:897-902.

Axtell RC, de Jong BA, Boniface K, van der Voort LF, Bhat R, De Sarno P, Naves R, Han M, Zhong F, Castellanos JG, Mair R, Christakos A, Kolkowitz I, Katz L, Killestein J, Polman CH, de Waal Malefyt R, Steinman L, Raman C. T helper type 1 and 17 cells determine efficacy of interferon-beta in multiple sclerosis and experimental encephalomyelitis.Nat Med. 2010;16:406-12.

Liu X, Leung S, Wang C, Tan Z, Wang J, Guo TB, Fang L, Zhao Y, Wan B, Qin X, Lu L, Li R, Pan H, Song M, Liu A, Hong J, Lu H, Zhang JZ. Crucial role of interleukin-7 in T helper type 17 survival and expansion in autoimmune disease.Nat Med. 2010;16:191-7.

Arima Y, Harada M, Kamimura D, Park JH, Kawano F, Yull FE, Kawamoto T, Iwakura Y, Betz UA, Márquez G, Blackwell TS, Ohira Y, Hirano T, Murakami M. Regional neural activation defines a gateway for autoreactive T cells to cross the blood-brain barrier. Cell. 2012;148:447-57.

Saijo K, Collier JG, Li AC, Katzenellenbogen JA, Glass CK. An ADIOL-ERβ-CtBP transrepression pathway negatively regulates microglia-mediated inflammation.Cell. 201; 145:584-95.

Seifert U, Bialy LP, Ebstein F, Bech-Otschir D, Voigt A, Schröter F, Prozorovski T, Lange N, Steffen J, Rieger M, Kuckelkorn U, Aktas O, Kloetzel PM, Krüger E. Cell. 2010 20;142:613-24.

Liu B, Tahk S, Yee KM, Fan G, Shuai K. The ligase PIAS1 restricts natural regulatory T cell differentiation by epigenetic repression. Science. 2010;330:521-5.

Alvarez JI, Dodelet-Devillers A, Kebir H, Ifergan I, Fabre PJ, Terouz S, Sabbagh M, Wosik K, Bourbonnière L, Bernard M, van Horssen J, de Vries HE, Charron F, Prat A. The Hedgehog pathway promotes blood-brain barrier integrity and CNS immune quiescence. Science. 2011 ;334:1727-31.

Okamoto K, Iwai Y, Oh-Hora M, Yamamoto M, Morio T, Aoki K, Ohya K, Jetten AM, Akira S, Muta T, Takayanagi H. IkappaBzeta regulates T(H)17 development by cooperating with ROR nuclear receptors. Nature. 2010;464:1381-5.

Ghoreschi K, Laurence A, Yang XP, Tato CM, McGeachy MJ, Konkel JE, Ramos HL, Wei L, Davidson TS, Bouladoux N, Grainger JR, Chen Q, Kanno Y, Watford WT, Sun HW, Eberl G, Shevach EM, Belkaid Y, Cua DJ, Chen W, O'Shea JJ. Generation of pathogenic T(H)17 cells in the absence of TGF-β signalling. Nature. 2010;467::967-71.

Berer K, Mues M, Koutrolos M, Rasbi ZA, Boziki M, Johner C, Wekerle H, Krishnamoorthy G. Commensal microbiota and myelin autoantigen cooperate to trigger autoimmune demyelination. Nature. 2011 ;479::538-41.

Bai L, Lennon DP, Caplan AI, DeChant A, Hecker J, Kranso J, Zaremba A, Miller RH.

Hepatocyte growth factor mediates mesenchymal stem cell–induced recovery in multiple sclerosis models. Nat Neurosci. 2012 ;15:862-70.

Locatelli G, Wörtge S, Buch T, Ingold B, Frommer F, Sobottka B, Krüger M, Karram K, Bühlmann C, Bechmann I, Heppner FL, Waisman A, Becher B. Primary oligodendrocyte death does not elicit anti-CNS immunity. Nat Neurosci. 2012 ;15:543-50. .

Dann A, Poeck H, Croxford AL, Gaupp S, Kierdorf K, Knust M, Pfeifer D, Maihoefer C, Endres S, Kalinke U, Meuth SG, Wiendl H, Knobeloch KP, Akira S, Waisman A, Hartmann G, Prinz M. Cytosolic RIG-I-like helicases act as negative regulators of sterile inflammation in the CNS. Nat Neurosci. 2011 ;15:98-106.

Supplementary Table 2. **Search results for statistical analysis of EAE Data in high impact factor journals.** Results of a Pubmed search using the term ‘*experimental encephalomyelitis’* during a time period between January 2010 and August 2012 in *Nature* journals, *Cell* and *Science*.
