# Supplementary material for: Two Years Later: Journals Are Not Yet Enforcing the ARRIVE Guidelines on Reporting Standards for Pre-Clinical Animal Studies
Source: PLoS Biol. 2014 Jan 7;12(1):e1001756. doi: 10.1371/journal.pbio.1001756 (PMC3883646; doi:10.1371/journal.pbio.1001756)
Supplement: Table S3 — Search results for analysis of reporting outcomes in EAE publications. Results of a PubMed search using the term “experimental encephalomyelitis” during a two-year period for PLOS journals before (29 June 2008–28 June 2010) and after (29 June 2010–28 June 2012) endorsement of the ARRIVE guidelines, and for Nature journals before (1 February 2009–31 January 2011) and after (1 February 2011–31 January 2013) endorsement of the ARRIVE guidelines. (DOC) [file pbio.1001756.s003.doc]

**Supplementary Table 3**

**PLOS Papers Pre ARRIVE**

Aharonowiz, M., Einstein, O., Fainstein, N., Lassmann, H., Reubinoff, B., & Ben-Hur, T. (2008). Neuroprotective effect of transplanted human embryonic stem cell-derived neural precursors in an animal model of multiple sclerosis. *PloS one*, *3*(9), e3145. doi:10.1371/journal.pone.0003145

Andersen, K. G., Butcher, T., & Betz, A. G. (2008). Specific immunosuppression with inducible Foxp3-transduced polyclonal T cells. *PLoS biology*, *6*(11), e276. doi:10.1371/journal.pbio.0060276

Andersson, A., Isaksson, M., Wefer, J., Norling, A., Flores-Morales, A., Rorsman, F., Kämpe, O., et al. (2008). Impaired autoimmune T helper 17 cell responses following DNA vaccination against rat experimental autoimmune encephalomyelitis. *PloS one*, *3*(11), e3682. doi:10.1371/journal.pone.0003682

Brandl, C., Ortler, S., Herrmann, T., Cardell, S., Lutz, M. B., & Wiendl, H. (2010). B7-H1-deficiency enhances the potential of tolerogenic dendritic cells by activating CD1d-restricted type II NKT cells. *PloS one*, *5*(5), e10800. doi:10.1371/journal.pone.0010800

Bruzzone, S., Fruscione, F., Morando, S., Ferrando, T., Poggi, A., Garuti, A., D’Urso, A., et al. (2009). Catastrophic NAD+ depletion in activated T lymphocytes through Nampt inhibition reduces demyelination and disability in EAE. *PloS one*, *4*(11), e7897. doi:10.1371/journal.pone.0007897

Hatterer, E., Touret, M., Belin, M.-F., Honnorat, J., & Nataf, S. (2008). Cerebrospinal fluid dendritic cells infiltrate the brain parenchyma and target the cervical lymph nodes under neuroinflammatory conditions. *PloS one*, *3*(10), e3321. doi:10.1371/journal.pone.0003321

Hu, W., Metselaar, J., Ben, L.-H., Cravens, P. D., Singh, M. P., Frohman, E. M., Eagar, T. N., et al. (2009). PEG minocycline-liposomes ameliorate CNS autoimmune disease. *PloS one*, *4*(1), e4151. doi:10.1371/journal.pone.0004151

Lavasani, S., Dzhambazov, B., Nouri, M., Fåk, F., Buske, S., Molin, G., Thorlacius, H., et al. (2010). A novel probiotic mixture exerts a therapeutic effect on experimental autoimmune encephalomyelitis mediated by IL-10 producing regulatory T cells. *PloS one*, *5*(2), e9009. doi:10.1371/journal.pone.0009009

Melzer, N., Meuth, S. G., Torres-Salazar, D., Bittner, S., Zozulya, A. L., Weidenfeller, C., Kotsiari, A., et al. (2008). A beta-lactam antibiotic dampens excitotoxic inflammatory CNS damage in a mouse model of multiple sclerosis. *PloS one*, *3*(9), e3149. doi:10.1371/journal.pone.0003149

Pluchino, S., Zanotti, L., Brambilla, E., Rovere-Querini, P., Capobianco, A., Alfaro-Cervello, C., Salani, G., et al. (2009). Immune regulatory neural stem/precursor cells protect from central nervous system autoimmunity by restraining dendritic cell function. *PloS one*, *4*(6), e5959. doi:10.1371/journal.pone.0005959

Pötzl, J., Botteron, C., Tausch, E., Pedré, X., Mueller, A. M., Männel, D. N., & Lechner, A. (2008). Tracing functional antigen-specific CCR6 Th17 cells after vaccination. *PloS one*, *3*(8), e2951. doi:10.1371/journal.pone.0002951

Rynda, A., Maddaloni, M., Ochoa-Repáraz, J., Callis, G., & Pascual, D. W. (2010). IL-28 supplants requirement for T(reg) cells in protein sigma1-mediated protection against murine experimental autoimmune encephalomyelitis (EAE). *PloS one*, *5*(1), e8720. doi:10.1371/journal.pone.0008720

Sun, X., Wang, X., Chen, T., Li, T., Cao, K., Lu, A., Chen, Y., et al. (2010). Myelin activates FAK/Akt/NF-kappaB pathways and provokes CR3-dependent inflammatory response in murine system. *PloS one*, *5*(2), e9380. doi:10.1371/journal.pone.0009380

Wüst, S., Tischner, D., John, M., Tuckermann, J. P., Menzfeld, C., Hanisch, U.-K., Van den Brandt, J., et al. (2009). Therapeutic and adverse effects of a non-steroidal glucocorticoid receptor ligand in a mouse model of multiple sclerosis. *PloS one*, *4*(12), e8202. doi:10.1371/journal.pone.0008202

Yadav, R., Bhowmick, S., Gorecki, P., O’Rourke, J., & Cone, R. E. (2010). Paradoxical effect of pertussis toxin on the delayed hypersensitivity response to autoantigens in mice. *PloS one*, *5*(8), e11983. doi:10.1371/journal.pone.0011983

**PLOS Papers Post ARRIVE**

Atkinson, W., Forghani, R., Wojtkiewicz, G. R., Pulli, B., Iwamoto, Y., Ueno, T., Waterman, P., et al. (2012). Ligation of the jugular veins does not result in brain inflammation or demyelination in mice. *PloS one*, *7*(3), e33671. doi:10.1371/journal.pone.0033671

Avnir, Y., Turjeman, K., Tulchinsky, D., Sigal, A., Kizelsztein, P., Tzemach, D., Gabizon, A., et al. (2011). Fabrication principles and their contribution to the superior in vivo therapeutic efficacy of nano-liposomes remote loaded with glucocorticoids. *PloS one*, *6*(10), e25721. doi:10.1371/journal.pone.0025721

Casiraghi, C., Shanina, I., Cho, S., Freeman, M. L., Blackman, M. a, & Horwitz, M. S. (2012). Gammaherpesvirus latency accentuates EAE pathogenesis: relevance to Epstein-Barr virus and multiple sclerosis. *PLoS pathogens*, *8*(5), e1002715. doi:10.1371/journal.ppat.1002715

Chang, J.-H., Cha, H.-R., Lee, D.-S., Seo, K. Y., & Kweon, M.-N. (2010). 1,25-Dihydroxyvitamin D3 inhibits the differentiation and migration of T(H)17 cells to protect against experimental autoimmune encephalomyelitis. *PloS one*, *5*(9), e12925. doi:10.1371/journal.pone.0012925

Doerck, S., Göbel, K., Weise, G., Schneider-Hohendorf, T., Reinhardt, M., Hauff, P., Schwab, N., et al. (2010). Temporal pattern of ICAM-I mediated regulatory T cell recruitment to sites of inflammation in adoptive transfer model of multiple sclerosis. *PloS one*, *5*(11), e15478. doi:10.1371/journal.pone.0015478

Domingues, H. S., Mues, M., Lassmann, H., Wekerle, H., & Krishnamoorthy, G. (2010). Functional and pathogenic differences of Th1 and Th17 cells in experimental autoimmune encephalomyelitis. *PloS one*, *5*(11), e15531. doi:10.1371/journal.pone.0015531

Dutra, R. C., Leite, D. F. P., Bento, A. F., Manjavachi, M. N., Patrício, E. S., Figueiredo, C. P., Pesquero, J. B., et al. (2011). The role of kinin receptors in preventing neuroinflammation and its clinical severity during experimental autoimmune encephalomyelitis in mice. *PloS one*, *6*(11), e27875. doi:10.1371/journal.pone.0027875

Farias, A. S., Talaisys, R. L., Blanco, Y. C., Lopes, S. C. P., Longhini, A. L. F., Pradella, F., Santos, L. M. B., et al. (2011). Regulatory T cell induction during Plasmodium chabaudi infection modifies the clinical course of experimental autoimmune encephalomyelitis. *PloS one*, *6*(3), e17849. doi:10.1371/journal.pone.0017849

Guo, X., Harada, C., Namekata, K., Mitamura, Y., Yoshida, H., Matsumoto, Y., & Harada, T. (2010). Delayed onset of experimental autoimmune encephalomyelitis in Olig1 deficient mice. *PloS one*, *5*(9), 1–11. doi:10.1371/journal.pone.0013083

Haque, A., Best, S. E., Amante, F. H., Mustafah, S., Desbarrieres, L., De Labastida, F., Sparwasser, T., et al. (2010). CD4+ natural regulatory T cells prevent experimental cerebral malaria via CTLA-4 when expanded in vivo. *PLoS pathogens*, *6*(12), e1001221. doi:10.1371/journal.ppat.1001221

Herges, K., Millward, J. M., Hentschel, N., Infante-Duarte, C., Aktas, O., & Zipp, F. (2011). Neuroprotective effect of combination therapy of glatiramer acetate and epigallocatechin-3-gallate in neuroinflammation. *PloS one*, *6*(10), e25456. doi:10.1371/journal.pone.0025456

Iken, S., Bachy, V., Gourdain, P., Lim, A., Grégoire, S., Chaigneau, T., Aucouturier, P., et al. (2011). Th2-polarised PrP-specific transgenic T-cells confer partial protection against murine scrapie. *PLoS pathogens*, *7*(9), e1002216. doi:10.1371/journal.ppat.1002216

Jia, Y., Jing, J., Bai, Y., Li, Z., Liu, L., Luo, J., Liu, M., et al. (2011). Amelioration of experimental autoimmune encephalomyelitis by plumbagin through down-regulation of JAK-STAT and NF-κB signaling pathways. *PloS one*, *6*(10), e27006. doi:10.1371/journal.pone.0027006

Kaushansky, N., Kerlero de Rosbo, N., Zilkha-Falb, R., Yosef-Hemo, R., Cohen, L., & Ben-Nun, A. (2011). “Multi-epitope-targeted” immune-specific therapy for a multiple sclerosis-like disease via engineered multi-epitope protein is superior to peptides. *PloS one*, *6*(11), e27860. doi:10.1371/journal.pone.0027860

Kim do, Y., Hao, J., Liu, R., Turner, G., Shi, F.D., &Rho, J.M. (2012). Inflammation-mediated memory dysfunction and effects of a ketogenic diet in a murine model of multiple sclerosis.*PLoS one*, 7(5):e35476.doi: 10.1371/journal.pone.0035476.

Ma, X., Jiang, Y., Wu, A., Chen, X., Pi, R., Liu, M., & Liu, Y. (2010). Berberine attenuates experimental autoimmune encephalomyelitis in C57 BL/6 mice. *PloS one*, *5*(10), e13489. doi:10.1371/journal.pone.0013489

Monson, N. L., Cravens, P., Hussain, R., Harp, C. T., Cummings, M., De Pilar Martin, M., Ben, L.-H., et al. (2011). Rituximab therapy reduces organ-specific T cell responses and ameliorates experimental autoimmune encephalomyelitis. *PloS one*, *6*(2), e17103. doi:10.1371/journal.pone.0017103

Muili, K. a, Gopalakrishnan, S., Meyer, S. L., Eells, J. T., & Lyons, J.-A. (2012). Amelioration of experimental autoimmune encephalomyelitis in C57BL/6 mice by photobiomodulation induced by 670 nm light. *PloS one*, *7*(1), e30655. doi:10.1371/journal.pone.0030655

Payne, N. L., Sun, G., Herszfeld, D., Tat-Goh, P. a, Verma, P. J., Parkington, H. C., Coleman, H. a, et al. (2012). Comparative study on the therapeutic potential of neurally differentiated stem cells in a mouse model of multiple sclerosis. *PloS one*, *7*(4), e35093. doi:10.1371/journal.pone.0035093

Saederup, N., Cardona, A. E., Croft, K., Mizutani, M., Cotleur, A. C., Tsou, C.-L., Ransohoff, R. M., et al. (2010). Selective chemokine receptor usage by central nervous system myeloid cells in CCR2-red fluorescent protein knock-in mice. *PloS one*, *5*(10), e13693.doi:10.1371/journal.pone.0013693

Schulze-Topphoff, U., Shetty, A., Varrin-Doyer, M., Molnarfi, N., Sagan, S. a, Sobel, R. a, Nelson, P. a, et al. (2012). Laquinimod, a quinoline-3-carboxamide, induces type II myeloid cells that modulate central nervous system autoimmunity. *PloS one*, *7*(3), e33797. doi:10.1371/journal.pone.0033797

Sinha, S., Miller, L. M., Subramanian, S., Burrows, G. G., Vandenbark, A. a, & Offner, H. (2011). RTL551 treatment of EAE reduces CD226 and T-bet+ CD4 T cells in periphery and prevents infiltration of T-bet+ IL-17, IFN-γ producing T cells into CNS. *PloS one*, *6*(7), e21868. doi:10.1371/journal.pone.0021868

Stridh, P., Thessen Hedreul, M., Beyeen, A. D., Adzemovic, M. Z., Laaksonen, H., Gillett, A., Ockinger, J., et al. (2010). Fine-mapping resolves Eae23 into two QTLs and implicates ZEB1 as a candidate gene regulating experimental neuroinflammation in rat. *PloS one*, *5*(9), e12716. doi:10.1371/journal.pone.0012716

Takata, K., Kinoshita, M., Okuno, T., Moriya, M., Kohda, T., Honorat, J. a, Sugimoto, T., et al. (2011). The lactic acid bacterium Pediococcus acidilactici suppresses autoimmune encephalomyelitis by inducing IL-10-producing regulatory T cells. *PloS one*, *6*(11), e27644. doi:10.1371/journal.pone.0027644

Vaknin, I., Kunis, G., Miller, O., Butovsky, O., Bukshpan, S., Beers, D. R., Henkel, J. S., et al. (2011). Excess circulating alternatively activated myeloid (M2) cells accelerate ALS progression while inhibiting experimental autoimmune encephalomyelitis. *PloS one*, *6*(11), e26921. doi:10.1371/journal.pone.0026921

Varthaman, A., Clement, M., Khallou-Laschet, J., Fornasa, G., Gaston, A.-T., Dussiot, M., Caligiuri, G., et al. (2011). Physiological induction of regulatory Qa-1-restricted CD8+ T cells triggered by endogenous CD4+ T cell responses. *PloS one*, *6*(6), e21628. doi:10.1371/journal.pone.0021628

Weber, M. S., Benkhoucha, M., Lehmann-Horn, K., Hertzenberg, D., Sellner, J., Santiago-Raber, M.-L., Chofflon, M., et al. (2010). Repetitive pertussis toxin promotes development of regulatory T cells and prevents central nervous system autoimmune disease. *PloS one*, *5*(12), e16009. doi:10.1371/journal.pone.0016009

Wu, M., Nissen, J.C., Chen, E.L., &Tsirka, S.E. (2012).Tuftsin Promotes an Anti-Inflammatory Switch and Attenuates Symptoms in Experimental Autoimmune Encephalomyelitis. *PLoSone*, 7(4):e34933. doi: 10.1371/journal.pone.0034933.

Yin, J., Tu, J., Lin, H., Shi, F., Liu, R., Zhao, C., Coons, S. W., et al. (2010). Centrally administered pertussis toxin inhibits microglia migration to the spinal cord and prevents dissemination of disease in an EAE mouse model. *PloS one*, *5*(8), e12400. doi:10.1371/journal.pone.0012400

Supplementary Table 3. **Search results for analysis of reporting outcomes in EAE publications**. Results of a Pubmed search using the term ‘*experimental encephalomyelitis’* during a 2-year period before (29 June 2008 – 28 June 2010) and after (29 June 2010 – 28 June 2012) and for *Nature* journals before (01 Feb 2009 – 31 Jan 2011) and after (01 Feb 2011 – Jan 31 2013) the initial endorsement of the ARRIVE guidelines in each respective journal.
