# Supplementary material for: Two Years Later: Journals Are Not Yet Enforcing the ARRIVE Guidelines on Reporting Standards for Pre-Clinical Animal Studies
Source: PLoS Biol. 2014 Jan 7;12(1):e1001756. doi: 10.1371/journal.pbio.1001756 (PMC3883646; doi:10.1371/journal.pbio.1001756)
Supplement: Text S1 — Supplementary methods and results. Search methods, statistical analysis, and reporting outcomes. (DOC) [file pbio.1001756.s004.doc]

**Text S1**

1. **Search strategy for EAE publications**

A search of *Pubmed* (www.pubmed.org) for manuscripts and e-publications containing the search term ‘*experimental encephalomyelitis’* was undertaken. This was selected to avoid issues of related to the use of ‘*autoimmune’* or ‘*allergic’* that is interchangeably used to describe EAE. The search was restricted to articles in English and a 6-month period between 1 December 2011 and 31 May 2012, was analysed following publication of suggested guidelines on reporting and statistical analysis of EAE data in relation to the ARRIVE guidelines [1]. It is acknowledged that this search method was not exhaustive as there are manuscripts in journals not included on *Pubmed*, furthermore it would perhaps take time for authors to adopt guidelines, but it provided an indication of the nature of experimental design. At the time of the search, 304 manuscripts were identified (Supplementary Table 1) and copies were obtained using library subscriptions or a reprint from the author. These were initially screened to exclude 86 manuscripts that were reviews, or were not concerned with EAE. An additional 38 manuscripts on EAE were eliminated, as they did not contain comparisons of clinical scores of two different groups. The remaining 180 manuscripts comparing EAE scores in two or more groups of animals were examined to determine whether inappropriate parametric tests (Analysis of Variance, Linear regression and/or t tests) [7] or appropriate non-parametric tests (Mann Whitney U and/or Kruskall Wallis tests) were applied. This evaluation was performed by two independent observers. Although arguable, we have taken a stance that it is statistically incorrect to apply parametric statistics to such neurological score data without data manipulation, such as ranking prior undertaking parametric statistics, although we accept that some statisticians could argue this case [7]. However, the studies were assessing the same hypothesis on the same type of data set and therefore one should expect some consistency.

1. **Statistical Analysis of EAE Data in the General Literature**

It was found that 23/180 (13%. 95% confidence Interval (CI) 8.7%-18.5%) manuscripts did not report statistical analyses of the comparison of groups, or did not report the type of statistics used to analyse the data. In addition, 5/180 manuscripts reported use of both types of statistics, but did not indicate to which experiments the tests applied to. Surprisingly, only 69/175 (39%. 95%CI 32.5%-46.8%) manuscripts reported the sole use of non-parametric statistics on non-parametric neurological scoring systems. Rather disturbingly, 83/175 (47%. 95%CI 40.2%-54.8%) manuscripts reported the use of parametric statistics on non-parametric neurological scores. While 38/83 (46%. 95% CI 35.5%-56.5%) of these studies used t-tests to compare maximal neurological scores or clinical scores over time, linear regression and a variety of Analysis of Variance (ANOVA) and post-hoc tests were also inappropriately applied. Based on the proportion of studies originating from different global regions, it was found that inappropriate statistical analysis was universally consistent when the Americas (56%), Europe (54%) and Asia and Oceania (52%) were analysed. The largest number of manuscripts on EAE during this period were published in the *Journal of Immunology* (n=23) and the *Journal of Neuroimmunology* (n=13), in which adequate, non-parametric statistics were reported in 39% and 31% of cases, respectively. This shows the inconsistency of refereeing and reporting within journals. In summary, of the 152 manuscripts in which the statistical methods were clearly described, as much as 55% (95%CI 46.7-62.3%) used inappropriate statistical analysis of data. It is therefore probable that an even higher proportion of manuscripts with inappropriate statistical methods were submitted, but corrected during the peer review process.

1. **Statistical Analysis of EAE Data in high impact factor journals**

To address whether the quality of refereeing was related to the perceived quality of the journals, the statistics used in a manuscript was related to the 2011 *Web of Science* impact factor of the journal in which it was published (Figure 1). This revealed that reporting of inappropriate statistics occurs throughout the range of high and low impact factor publications [Figure 1]. Indeed, compared to 3/69 (95% CI 1.5%-12.0%) publications with correct reporting in journals that had an impact factor >10, almost double that number, i.e. 10/107 (95%CI 5.2%-16.4%) publications in those journals used incorrect statistics, or failed to report the statistics. To examine this further, manuscripts published from 1 January 2010 to August 2012 in *Nature Neuroscience, Nature Medicine*, *Nature Immunology*, *Cell* and *Science* were examined using the search terms of *experimental* *encephalomyelitis* and *multiple sclerosis* identified (Supplementary Table 2). The use of the term “multiple sclerosis” was used to capture EAE papers that we knew existed by were not found with the former search terms, to keep the sample size as high as possible. These were again separated into those that examined neurological scores in at least two groups of animals. It was again revealing that only 1/26 (4%. 95%CI 0.7-18.9%) manuscripts reported the adequate sole use of non-parametric analysis of data on neurological scores, 3/26 reported both non-parametric and parametric analysis, while 15/26 (57.7%. 95% CI 40.0-74.5%), used parametric statistics. Of these, 10/15 (67%. 95%CI 41.7%-84.8%) only used the t test, as many as 7/26 (27%) did not report the type of statistics used, or did not use any statistical analysis at all, despite claiming effects by a certain treatment or gene knockout.

1. **Reporting Outcomes in the EAE in ARRIVE guideline-endorsing Journals**

The original study on one issue of experimental design, which relates to one point of the ARRIVE guidelines, led us to wonder, whether other aspects of the ARRIVE guidelines were being adopted. The introduction of the ARRIVE guidelines, which were first published in *PLOS Biology* in June 2010, aimed to improve such reporting standards [3]. These have been adopted by an increasing number of charities as part of their funding requirements and have been endorsed by approximately 200 journals, which notably include the *Nature* and *PLOS* journals. We again restricted the survey to studies on EAE and restricted this to two prominent endorsees of the ARRIVE guidelines. A literature search of *PLOS* journals was performed for a 2-year period before (29 June 2008 – 28 June 2010) and after (29 June 2010 – 28 June 2012) and for *Nature* journals before (01 Feb 2009 – 31 Jan 2011) and after (01 Feb 2011 – Jan 31 2013) the initial publication/endorsement of the ARRIVE guidelines in each respective journal. Manuscripts containing the initial search term ‘*experimental encephalomyelitis’,* and did not include ‘*multiple sclerosis’* that detected too many irrelevant papers*,* and reporting a comparison of neurological disease score were included in the survey. It was found that there were a number of EAE papers before (n=15, *PLOS journals*; n=15, *Nature journals*) and after (n=30, *PLOS Journals essentially all in PLOS One*; n=14 *Nature Journals.* identified (Supplementary Table 3) the publication of ARRIVE guidelines. Manuscripts were evaluated in four key areas that were selected based upon the comprehensive survey undertaken prior to the introduction of ARRIVE [4]. The areas chosen included ethics, study design (allocation to groups/randomisation and blinding), experimental animals (species, sex, age and group size) and sample size estimation/power calculations (Figure 2). These encompassed elements of suggested core reporting requirements [5]. We did not assess all 20 outcomes of ARRIVE guidelines, previous studies have suggested that no/very few papers fully incorporate all aspects of the ARRIVE guidelines [6].

Ethical review statements have now become a common statement feature requested by journals and was a commonly-reported feature in both *PLOS* (93% pre-ARRIVE, 94% post-ARRIVE), *Nature* manuscripts (100% pre-ARRIVE, 100% post-ARRIVE) and other journals [7]. However, methods to reduce bias and the chance of false-positive reporting, are poorly reported, although it does not mean they are not part of experimental design [2, 9]. It was previously reported that 86-87% of animal studies, including EAE studies did not report on issues of randomization and blinding [4, 7]. Analysis of manuscripts accepted after the publication of ARRIVE demonstrated that both *PLOS* and *Nature* journals published a very small proportion of manuscripts reporting blinding (20% and 21%, respectively) and randomization (10% and 0%, respectively). This results in over-estimation of treatment effects, which can impact on the translational value of the studies. Whilst it is accepted that some of these studies examined here may have been designed before the introduction of the ARRIVE guidelines, that ARRIVE is simply a reporting guideline should therefore not have precluded appropriate reporting had the journals adopted the standard on initial publication of the guidelines and provided the space the documentation of such information. However, most of the papers analyzed in PLOS journals were published after 2010. The guidelines indicated that authors should report basic parameters, which will facilitate replication of experimental results [7]. It is known that that animal characteristics (species, sex and age) and number of animals used in a study can potentially influence experimental outcomes. There was an increase in the reporting of species (100%, *PLOS journals*; 100% *Nature journals*), sex (68% *PLOS journals;* 79% *Nature journals*) and age (87% *PLOS*; 79% *Nature*) of animals following ARRIVE guidelines. However, not all manuscripts reported these simple data requirements (Figure 2). Likewise, in contrast to human trials where power calculations are an inherent part of undertaking clinical trials, this is rarely reported in animal studies [4, 7]. None of the studies in the PLOS journals reported power calculations and only 1/16 Nature journals before and 1/14 after the introduction of ARRIVE, reported power calculations (Supplementary Figure 2). There is nothing in the ARRIVE guidelines that compels researchers to analyse their data in a particular way, except to indicate that it is appropriate. As such reporting of statistical analysis was common, but as mentioned previously use of parametric statistics on non-parametric data was the norm and application of non-parametric statistics to neurological score data occurred less in *Nature* Journals (25% pre ARRIVE; 7% post ARRIVE).

**References**

1. Baker D & Amor S. (2012) Publication guidelines for refereeing and reporting on animal use in experimental autoimmune encephalomyelitis. J Neuroimmunol. 242: 78-83.

2. Flemming KK, Bovaird JA, Mosier MC, Emerson MR, Le Vine SM et al. (2005) Statistical analysis of data from studies on experimental autoimmune encephalomyelitis. J. Neuroimmunol. 170: 71-84.

3. Kilkenny C, Browne WJ, Cuthill IC, Emerson M & Altman DG. (2010) Improving bioscience research reporting: the ARRIVE guidelines for reporting animal research. PLoS Biol. 2010; 8(6): e1000412.

4. Kilkenny C, Parsons N, Kadyszewski E, Festing MFW, Cuthill IC, et al. (2009) Survey of the quality of experimental design, statistical analysis and reporting of research using animals. PLoS One 4(11): e7824.

5. Landis SC, Amara SG, Asadullah K, Austin CP, Blumenstein R, et al. (2012). A call for transparent reporting to optimize the predictive value of preclinical research. Nature. 490:187-91.

6. Schwarz F, Iglhaut G, Becker J. (2012) Quality assessment of reporting of animal studies on pathogenesis and treatment of peri-implant mucositis and peri-implantitis. A systematic review using the ARRIVE guidelines. J Clin Periodontol. 39 Suppl 12:63-72.

7. Vesterinen HM, Sena ES, ffrench-Constant C, Williams A, Chandran S, et al. (2010) Improving the translational hit of experimental treatments in multiple sclerosis. Mult Scler. 16: 1044-1055.
